# A synthesis of hydroclimatic, ecological, and socioeconomic data for transdisciplinary research in the Mekong

**DOI:** 10.1038/s41597-023-02193-0

**Published:** 2023-05-15

**Authors:** Amar Deep Tiwari, Yadu Pokhrel, Daniel Kramer, Tanjila Akhter, Qiuhong Tang, Junguo Liu, Jiaguo Qi, Ho Huu Loc, Venkataraman Lakshmi

**Affiliations:** 1 Department of Civil and Environmental Engineering, Michigan State University, East Lansing, Michigan USA; 2 Department of Fisheries and Wildlife, Michigan State University, East Lansing, Michigan USA; 3 Key Laboratory of Water Cycle and Related Land Surface Processes, Institute of Geographic Sciences and Natural Resources Research, Chinese Academy of Sciences, Beijing, China; 4 School of Water Conservancy, North China University of Water Resources and Electric Power, Zhengzhou, China; 5 Center for Global Change and Earth Observations, Michigan State University, East Lansing, Michigan USA; 6 Water Engineering and Management, Asian Institute of Technology, Khlong Nueng, Pathum Thani Thailand; 7 Engineering Systems and Environment, University of Virginia, Charlottesville, Virginia USA

**Keywords:** Hydrology, Environmental impact

## Abstract

The Mekong River basin (MRB) is a transboundary basin that supports livelihoods of over 70 million inhabitants and diverse terrestrial-aquatic ecosystems. This critical lifeline for people and ecosystems is under transformation due to climatic stressors and human activities (e.g., land use change and dam construction). Thus, there is an urgent need to better understand the changing hydrological and ecological systems in the MRB and develop improved adaptation strategies. This, however, is hampered partly by lack of sufficient, reliable, and accessible observational data across the basin. Here, we fill this long-standing gap for MRB by synthesizing climate, hydrological, ecological, and socioeconomic data from various disparate sources. The data— including groundwater records digitized from the literature—provide crucial insights into surface water systems, groundwater dynamics, land use patterns, and socioeconomic changes. The analyses presented also shed light on uncertainties associated with various datasets and the most appropriate choices. These datasets are expected to advance socio-hydrological research and inform science-based management decisions and policymaking for sustainable food-energy-water, livelihood, and ecological systems in the MRB.

## Background & Summary

### Features of the MRB

The Mekong is an important transboundary river that supports water supplies, fisheries, irrigated agriculture, energy generation, riverine ecosystems, navigation, and recreation in the six countries that it flows across: China, Myanmar, Laos, Thailand, Cambodia, and Vietnam^1–3^. An important ecological phenomenon characterized by a strong seasonal flow pattern that supports periodic inundation, as well as the exchange of water and nutrients between rivers and neighboring floodplains, known as the flood pulse^4^, is a lifeline to the numerous ecosystems services that the Mekong River basin (MRB) provides. In particular, the Tonle Sap Lake (TSL) and Mekong Delta are two important socio-ecological systems that rely on the Mekong flood pulse and support critical river-floodplain ecosystems as well as agriculture—especially rice farming—and fisheries^5–7^. The lake located in the Cambodian portion of the basin is home to one of the world’s most diverse inland fishery systems (e.g., Ziv *et al*.^8^). The fisheries and flood-recession agriculture in and around the lake are supported by the two-way flow in the Tonle Sap River^9^, driven by the Mekong flood pulse^5^. Furthermore, the Mekong Delta in Vietnam is a densely populated region and the third largest Delta in the world^10^. It is highly fertile and intensively cultivated, supporting the production of 7–10% of all rice traded worldwide^11^.

### Shifting hydrological and ecological systems

The natural synchronization among hydrology, flood pulse, fishery, agriculture, and riverine ecosystems has remained relatively stable for generations, but these tightly connected systems are being transformed in recent times due to climate change and growing human interventions^12–16^. In particular, climatic shifts, land use land cover changes, and a boom in hydropower dams have altered the river’s hydrology and ecology^9,17–19^, with profound implications on ecosystem services^20,21^, nutrient and sediment budgets^22^, and biodiversity and productivity^23,24^. Most notably, the construction of a series of dams along the mainstream Mekong is the dominant factor leading to a rapid transformation in the hydrological regime^25,26^. The effects of new dams also extend beyond hydrology and ecology, causing widespread changes in greenhouse gas (GHG) emissions, nutrient dynamics, and local livelihoods^24,27–31^.

As dam construction continues to accelerate across the MRB with increasing downstream impacts on food-energy-water (FEW) and ecological systems, there has been a growing interest in studying these consequences and developing improved adaptation strategies^32–36^. Data collected either on the ground or remotely, and models are indispensable tools for examining the nexus of these human activities, impacts and social implications, and providing reliable future scenarios. As such, numerous studies in the MRB have attempted to investigate these ongoing changes using observed data^3,37–40^, remote sensing products^1,16,41–43^, and process-based models^41,44–53^.

### Studies based on observed datasets

Using observed hydrometeorological and climate datasets in the MRB, several studies have investigated the role and implications of long-term climate change, short-term climate variability, and human activities on the FEW nexus. For example, Fan and He^37^ quantified the trends in precipitation and temperature to examine their effect on streamflow in the MRB. Mohammed *et al*.^41^ explored recent extreme flow events at various locations in the MRB. Other studies have examined the changes in groundwater, especially over the Mekong Delta or other small regions within the MRB, including those^46,54–56^ that have focused on groundwater analysis in the different parts of the basin. Numerous other studies have examined various aspects of hydrology and climatology over some parts of the basin^13,39,41,42,47,57,58^ which provide a small-scale or sub-basin level understanding of spatial and temporal variability. Associated nutrient dynamics have also been explored in a number of studies. Binh *et al*.^34^, for example, identified the decreasing trend in sediment in the Mekong Delta due to upstream dam construction and Whitehead *et al*.^59^ quantified the changes in nitrogen and phosphorus concentrations and fluxes. These studies have provided insights into many emerging issues but have also underscored data limitations that hindered more comprehensive, and basin-scale studies on the rapidly changing hydrological, agricultural, and ecological systems. Indeed, for the MRB, observational datasets are very limited, non-continuous, sparse, or not easily accessible.

### Studies based on remote sensing datasets

Some of the gaps and limitations in the ground-based observational data have been addressed using emerging data from satellite remote sensing. These data have been specifically used to characterize the changes in land use^1,43,60–63^, cropping patterns^64–66^, map open surface water areas^53,58,67–69^, develop dam attributes^67,70^, and landslide estimation^71^ among others. Further, remotely sensed precipitation data have been used as inputs in modeling studies^40,44,72^. Data from the Gravity Recovery and Climate Experiment (GRACE) satellite mission have been used to evaluate simulated surface storage, filling the gap in publicly available groundwater data for the MRB^9,44,73^. Remote sensing products have also been used to evaluate model results, filling the gaps in observational datasets, or providing added opportunities for model development and validation^45,50^. Despite these applications, remote sensing products suffer from inherent uncertainties resulting from numerous technical and methodological issues such as cloud contamination^67,74^. Further, while remote sensing products are spatially extensive, their temporal coverage and frequency for many products are limited.

### Gaps and Opportunities

The studies based on observed and remote sensing datasets have advanced the understanding of the processes, impacts, and drivers of hydrological and ecosystem shifts, especially in relation to dam construction^9,25,45,47,75–78^. However, they have also revealed critical data gaps that have hindered a more complete understanding of the many rapidly emerging issues. Ground-based observational data are either limited or not easily accessible for the MRB^79^. Satellite products can fill certain gaps but are not always reliable in terms of data quality and temporal gaps. Further, models can provide spatially-complete and temporally-continuous information, but the lack of observational data is a critical hurdle in constraining and validating process-based models. Compared to other global regions such as North America and Europe, data availability for the MRB is very limited^80^. Some data are available but there is no formal mechanism to share, are not easily accessible, or sharing is constrained due to institutional restrictions. Therefore, there are opportunities to bring light to the available data and their utility for increasingly important transdisciplinary research and collaboration^81,82^.

This paper fills a long-discussed and critical knowledge gap in data availability, accessibility, and utilization for the MRB. In this study, a synthesis of climate, hydrological, ecological, and socioeconomic datasets is provided, focusing on accuracy and utility, to guide the research community to the most suitable and reliable datasets for different purposes. Specifically, we synthesize and archived publicly available data using online repositories. For the datasets that are not publicly available (e.g., those available for purchase or through formal agreements), we identified the sources and presented the findings in graphical form, where possible. The key contribution of the paper lies in that it brings together many disparate datasets for hydrological, ecological, agricultural, and socioeconomic studies in the MRB, which are not readily available, not easily accessible, or are only available in graphical form in the published literature. It is expected that the paper could serve as a one-stop shop for certain key datasets for the aforementioned studies, which are increasingly essential to address growing issues on food, energy, water, and environmental systems across the MRB.

The rest of this paper is organized as follows. Methods section provides a detailed description of the numerous datasets, their sources, and methods to collect and produce the data. We discuss how to make use of disparate datasets that are freely available from their providers. Datasets that are not publicly available but are obtainable from the authors or agencies are also summarized. A list of data records is mentioned in the following section. In the technical validation section, we compare various datasets for each data type and provide insights on quality and validity. Based on the findings from this section, we provide usage notes for the available datasets in the last section.

## Methods

### Meteorological data

Meteorological datasets (e.g., precipitation, temperature, wind speed, relative humidity, sunshine hours; Table 1) are important elements of climate, hydrological, and ecosystems studies. These datasets constitute key inputs in hydrological modeling. Among various meteorological variables, precipitation serves as an entry point for a variety of applications relating to climate variability, hydrological modeling, agriculture, and ecosystems, among others^83,84^. In the MRB, precipitation measurements—along with other meteorological variables—are available at certain gauging stations but the placement of these stations is rather sparse and non-homogenous^85–87^. Various other meteorological products, especially for precipitation, are available that help fill observational data gaps and provide spatially continuous data for the entire MRB. For example, the Asian Precipitation-Highly Resolved Observational Data Integration Towards Evaluation (APHRODITE) product^88,89^, the only available long-term regional gauge-based daily gridded precipitation dataset for Asia^90^, is a key reference data used in many MRB studies^90–92^. Further, the Climate Hazards Group InfraRed Precipitation with Station data (CHIRPS; Funk *et al*.^93^), a 35 + year quasi-global rainfall dataset derived from gauge observations and satellite-based products and have recently been used by some MRB studies (e.g., Luo *et al*.^94^; Guo *et al*.^95^). Moreover, in the past three decades, earth observations by many satellite missions have provided spatially-complete and relatively high-resolution precipitation products, which have been increasingly used for many applications in the MRB^41,96–98^. Recent studies have compared these various precipitation datasets^40,44,72^, some suggesting that APHRODITE is amongst the most reliable precipitation products for the MRB^40,72^.

**Table 1:** Key variables and their primary sources.

S. No.	Data	Source	Native resolution	Data type	Remarks or weblinks*
1	Precipitation	EM-Earth	0.1°	Satellite	https://www.frdr-dfdr.ca/repo/dataset/8d30ab02-f2bd-4d05-ae43-11f4a387e5ad
2	Temperature	EM-Earth	0.1°	Satellite	https://www.frdr-dfdr.ca/repo/dataset/8d30ab02-f2bd-4d05-ae43-11f4a387e5ad
3	Wind Speed	MRC	Point data	*In Situ*	https://portal.mrcmekong.org
4	Relative Humidity	MRC	Point data	*In Situ*	https://portal.mrcmekong.org
5	Sunshine Hours	MRC	Point data	*In Situ*	https://portal.mrcmekong.org
6	Streamflow	MRC	Point data	*In Situ*	https://portal.mrcmekong.org
		Published literature	Point data	*In Situ*	Multiple sources
7	Water level	MRC	Point data	*In Situ*	https://portal.mrcmekong.org
8	Evapotranspiration	GLEAM	0.25°	Hybrid	https://www.gleam.eu/
		MODIS	500 m	Satellite	https://modis.gsfc.nasa.gov/data/dataprod/mod16.php
9	Surface water	Landsat based	30 m	Satellite	https://global-surface-water.appspot.com/download
		MODIS based	500 m	Satellite	http://data.starcloud.pcl.ac.cn/resource/9
10	Soil Moisture	SMAP	1 km	Satellite	https://nsidc.org/data/nsidc-0779/versions/1^133^
		Ground-based	Point data	*In Situ*	Collected by an individual scholar and his team
11	Groundwater	Published literature	Point data	*In Situ*	Multiple sources
12	Dam data	WLE	Point data	*In Situ*	https://wle-mekong.cgiar.org/
13	Nutrients	MRC	Point data	*In Situ*	https://portal.mrcmekong.org
14	Sediment Concentration	MRC	Point data	*In Situ*	https://portal.mrcmekong.org
15	Land use	ESA-CCI	300 m	Satellite	https://www.esa-landcover-cci.org/
16	Crop yield	FAOSTAT	Country-wise	*In Situ*	https://www.fao.org/faostat/en/#data/QCL
17	Crop calendar	GGCMI	0.5°	Hybrid	https://zenodo.org/record/5062513#.ZBHs5nrMIuU
18	Irrigated area and Irrigation water use	FAO	0.0833°	Satellite	https://www.fao.org/aquastat/en/geospatial-information/global-maps-irrigated-areas/latest-version
19	Irrigation water use (Cambodia)	Census	Point data	*In Situ*	Available for Cambodia
20	Wetlands	SWAMP	123 m	Satellite	https://www2.cifor.org/swamp/
		Tootchi *et al*.^192^	500 m	Satellite	https://doi.pangaea.de/10.1594/PANGAEA.892657
21	Inundation maps	Fluet-Chouinard *et al*.^193^	500 m	Satellite	www.estellus.fr/index.php?static13/giems-d15
22	Greenhouse gases	(EDGAR) v4.3.2	0.1°	Satellite	https://edgar.jrc.ec.europa.eu/
23	Socio-economic	Census	-	Hybrid	Multiple sources
24	Population	SEDAC	30-arc second	Hybrid	https://sedac.ciesin.columbia.edu/data/collection/gpw-v4
25	Population projections	Jones & O’Neill^212^	1/8°	Hydrid	http://sedac.ciesin.columbia.edu/data/set/popdynamics-1-8th-pop-base-year-projection-ssp-2000-2100-rev01/data-download)
26	GDP	DRYAD	30-arc second	*In Situ*	https://datadryad.org/stash/dataset/doi:10.5061/dryad.dk1j0
27	GDP projections	Wang & Sun^207^	0.25°	Hybrid	10.5281/zenodo.5880037

*These are the weblinks of the raw datasets.

As such, precipitation products are seemingly many; however, their spatial resolution and temporal availability period limit their utility for many applications; for example, process-based hydrological modeling often requires sub-daily data (e.g., Kabir *et al*.^44^), but many products noted above include only daily datasets. Table 2 summarizes, to our knowledge, the existing precipitation products, mostly global, with details on their resolution and availability period.

**Table 2:** Summary of various precipitation products useful for MRB studies.

S. No.	Data	Period	Spatial resolution	Temporal resolution	Data type	Reference
1	Mekong River Commission (MRC)	Station-dependent	Point data	Daily	Observed	—
2	Asian Precipitation-Highly Resolved Observational Data Integration Towards Evaluation (APHRODITE)	1951–2015	0.25°	Daily	Satellite	Yatagai *et al*.^88,89^
3	Climate Hazards Group InfraRed Precipitation with Station data (CHIRPS)	1981-present	0.05°	Daily	Satellite	Funk *et al*.^93^
4	Tropical Rainfall Measuring Mission (TRMM)	1998–2019	0.25°	3 hourly	Satellite	Simpson *et al*.^226^
5	Global Precipitation Measurement (GPM)	2014-present	0.1°	3 hourly	Satellite	Hou *et al*.^227,228^
6	Integrated Multi-satellitE Retrievals for GPM (IMERG)	2000-present	0.1°	30 minutes	Satellite	Huffman *et al*.^229^
7	TRMM Multi-satellite Precipitation Analysis (TMPA)	1998–2019	0.25°	3 hourly	Satellite	Huffman *et al*.^230^
8	Climate Prediction Center Morphing (CMORPH)	1998–present	8 km	30 minutes	Satellite	Joyce *et al*.^231^
9	Precipitation Estimation from Remotely Sensed Information using Artificial Neural Networks (PERSIANN)	1983-present	0.25°	Daily	Satellite	Hsu *et al*.^232^; Sorooshian *et al*.^233^
10	Climate Prediction Center (CPC)	1979-present	0.5°	Daily	Satellite	Xie *et al*.^234^
11	Climate Research Unit (CRU)	1901–2018	0.5°	Monthly	Satellite	Harris *et al*.^235^
12	Global Precipitation Climatology Center (GPCC)	1891-present	0.5°	Monthly	Satellite	Schneider *et al*.^236^
13	Precipitation Reconstruction over Land (PREC/L)	1948-present	0.5°	Monthly	Satellite	Chen *et al*.^237^
14	Multi-Source Weighted-Ensemble Precipitation v1.0 (MSWEP)	1979-present	0.25°	Daily	Satellite	Beck *et al*.^238^
15	ECMWF Reanalysis v5 (ERA5)	1959-present	0.25°	Hourly	Satellite	Hersbch *et al*.^99^
16	Princeton data	1948–2016	0.25°	Daily	Hybrid	He *et al*.^100^
17	TerraClimate	1958–2015	1/24°	Monthly	Hybrid	Abatzoglou *et al*.^239^
18	EM-Earth	1950–2019	0.1°	Daily	Hybrid	Tang *et al*.^110^

**Fig. 1:**
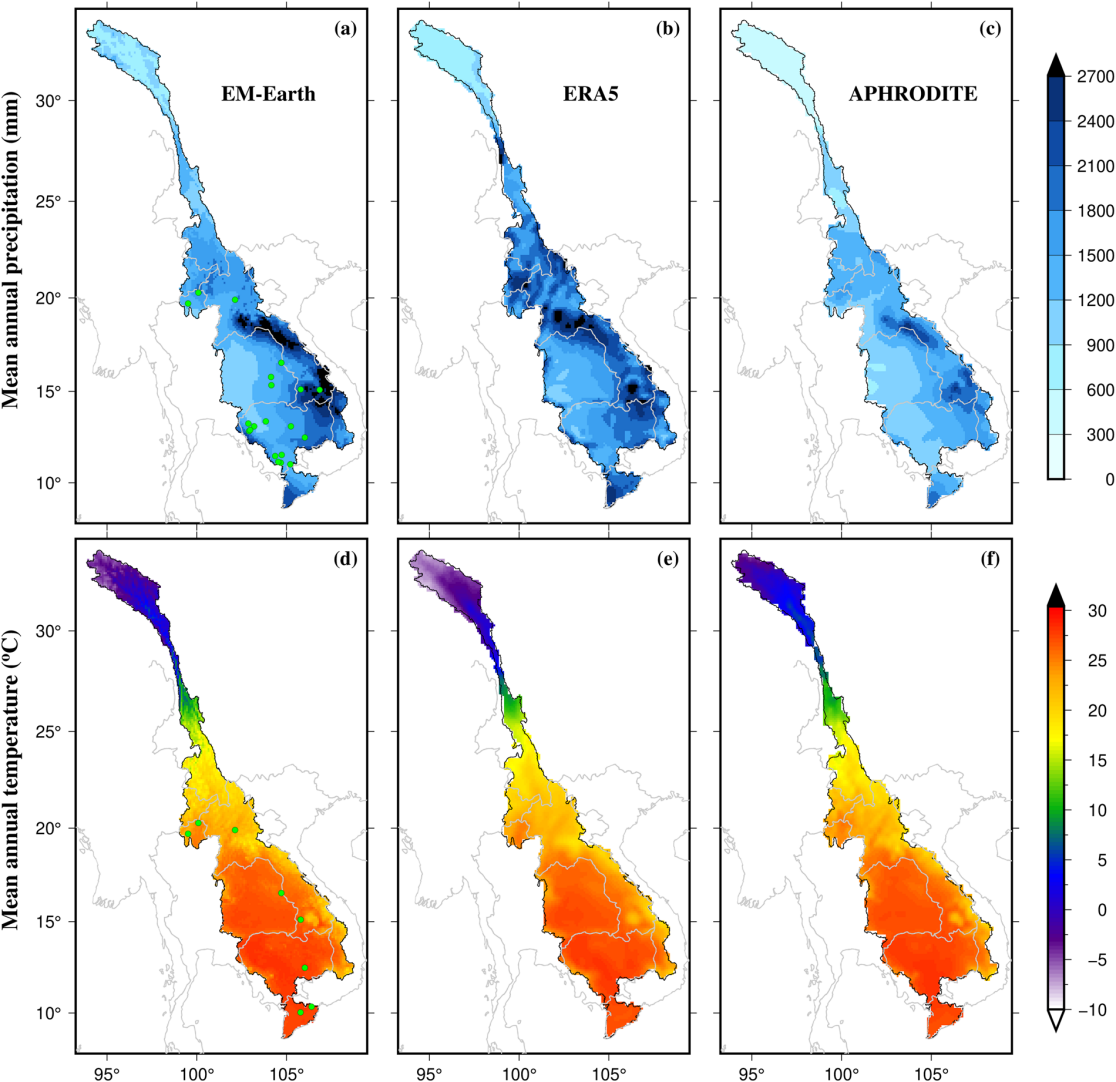
Spatial coverage of mean annual precipitation (mm) (1971–2000) for (**a**) EM-Earth, (**b**) ERA5, and (**c**) APHRODITE data and mean annual temperature (°C) (1971–2000) for (**a**) EM-Earth, (**b**) ERA5, and (**c**) APHRODITE data. Green circles in precipitation and temperature data panels (**a** and **d**) show gauging stations for observed datasets obtained from the MRC.

Many studies, especially on hydrological modeling, require meteorological input other than precipitation, which includes temperature, solar radiation, humidity, surface pressure, and wind speed. Such data are largely lacking for the MRB, except for the sparse gauge-based data from the MRC (Fig. 1). Therefore, modeling studies generally employ data from global products, which are primarily based on atmospheric reanalysis such as the ECMWF Reanalysis v5 (ERA5; Hersbach *et al*.^99^). There are numerous other global products that could be used for basin-scale modeling, which are derived from different reanalysis datasets. These include the Princeton Global Forcing data^100,101^, WATCH Forcing methodology applied to ERA-Interim reanalysis data (WFDEI^102,103^), meteorological forcing data of the third Global Soil Wetness Projects (GSWP3; Kim^104^), and WFDEI5 over land merged with ERA5 over the ocean (W5E5; Lange *et al*.^105^). Brocca *et al*.^106^ proposed an algorithm to estimate the effective rainfall data from *in-situ* soil moisture data (SM2RAIN). Later, this algorithm has been applied to various satellite-based soil moisture datasets to estimate the global effective rainfall (e.g., SM2RAIN-CCI (Ciabatta *et al*.^107^), SM2RAIN–ASCAT (Brocca *et al*.^108^), and GMP + SM2RAIN (Massari *et al*.^109^)). One common limitation in many of these products is the coarse spatial resolution (typically 0.5° ~50 km at the equator), which limits the application to only basin-scale modeling studies^19,44^.

To overcome the limitations related to spatial-temporal resolution and inherent biases, recent efforts have led to the development of higher resolution products such as the Ensemble Meteorological Dataset for Planet Earth (EM-Earth) data at 0.1° (~10 km at the equator) spatial resolution over global land areas from 1950 to 2019^110^. These data have 25 ensemble members enabling uncertainty analyses and sensitivity test in hydrological modeling. Another such recent product is the Climatologies at high resolution for the earth’s land surface areas (CHELSA) data (Karger *et al*.^111^; https://chelsa-climate.org/), also available at 30 arc seconds (~1 km) globally. However, both of these products are available at a daily time step, limiting the utility to models that only resolve water balance; land surface models that resolve energy balance typically require sub-daily datasets^112^. Nevertheless, the EM-Earth ensemble datasets have the potential to be useful for probabilistic climate and hydrological modeling. We have synthesized these datasets or have noted relevant sources where data are readily accessible.

### Hydrological data

#### Streamflow and water level

The primary source of the observed hydrological data in the MRB is the Mekong River Commission (MRC), which provides gauge-based data on river discharge and water levels at over 29 and 47 stations for streamflow and water level, respectively, across the basin (available through formal agreement or for purchase). Water level observations are also available from other sources such as the Cambodia Ministry of Water Resources and have been presented in the published literature (e.g., Arias *et al*.^113^). Observed data for the Chinese portion of the basin (i.e., Upper MRB (UMRB)) are generally not available to the international community but have been presented in some journal articles^3,26,37,114,115^. Some disparate streamflow data also exists for selected stations including Yun Jing Hong/ Yunjinghong^114,116^ (Chinese border), Jiuzhou^37,116^, Gajiu^116^, and Changdu^117^ stations in China; we have digitized these from the published literatures. The Global Runoff Data Center (GRDC) provides some streamflow data for the MRB within its global database but only for a small number of stations, which are included in the MRB database. Here, we present the complete information on available data from the MRB and other sources, along with some infographics.

#### Evapotranspiration (ET)

Similarly, ET is typically not measured *in-situ* due to the difficulty of deploying a network of ground-based networks over the entire area in the MRB. Therefore, satellite-based ET products, which provide a continuous record of ET at a global scale with a relatively high temporal resolution, are often used as an alternative. Some global ET products that have been used in the MRB include the water balance (WB; Zeng *et al*.^118^) based ET, GLEAM product (Martens *et al*.^119^), Penman-Monteith-Leuning data (PMLv2; Zhang *et al*.^120^) (500 m), Moderate-resolution Imaging Spectroradiometer global terrestrial evapotranspiration product (MOD16; Mu *et al*.^121^) (500 m), and Global LAnd Surface Satellite datasets (GLASS; Xie *et al*.^122^) (1 km). Studies such as Hu and Mo^123^ and Chen *et al*.^124^ have evaluated the performance of these products in the MRB and found that their accuracy can vary depending on specific conditions and characteristics of the region. Chen *et al*.^124^ concluded that Moderate Resolution Imaging Spectroradiometer (MODIS) ET underperforms in the MRB compared to other selected datasets. While Hu and Mo^123^ compared model simulated ET with satellite datasets and suggested that, in the MRB, GLEAM ET performs comparatively better than other products. Further, MODIS ET does not include data for land cover types specified as unclassified, urban, wetlands, perennial snow/ice, and permanent water bodies. Here, given certain uncertainties in both GLEAM (version 3.6b) and MODIS (version 6.1, gap filled) ET datasets and lack of observational data, we compare the two to demonstrate how they differ spatially and temporally.

#### Surface water

Monitoring surface water volume is a crucial aspect of water resource management, as it helps understand the availability and dynamics of water in a region. Surface water can be monitored using surface water area and water level^125,126^. Satellite altimetry datasets, such as those provided by TOPEX/Poseidon, Jason-1, Jason-2, Jason-3, and Envisat, use radar measurements to determine the height of the water surface and satellite imagery, such as that provided by MODIS, LANDSAT, and Sentinel, can be used to measure surface water area. For example, European Commission’s Joint Research Centre (JRC), developed by Pekel *et al*.^127^ used LANDSAT data at a spatial resolution of 30 meters to monitor surface water extent from 1984 to 2015. However, the temporal resolution of these datasets is relatively coarse, and they are available only in the form of percentage water occurrence at the monthly scale or as yearly classification. Moreover, there are limited images only for MRB which are cloud-free^9^. The recently launched Surface Water and Ocean Topography (SWOT) mission is expected to enable us address some of these limitations and greatly improve our ability to monitor surface water volume, especially by providing high-resolution data on surface water area and water level. Further, there are other satellite-based surface water products such as those generated by Ji *et al*.^128^ using MODIS data, which are available at the daily interval at the spatial resolution of 500 m and for the 2001–2016 period (data source: http://data.starcloud.pcl.ac.cn/resource/9). Here, we have processed and compared the two remotely sensed surface water products by Pekel *et al*.^127^ and Ji *et al*.^128^ for MRB, and present the processed surface water datasets to the community.

#### Soil moisture

*In-situ* soil moisture data for the MRB are limitedly available, if not non-existent at the basin-scale. As a result, the only choice is to use globally available remote sensing-based soil moisture products. For example, soil moisture data are available from the i) Soil Moisture Active Passive (SMAP; Entekhabi *et al*.^129^) at 9 km spatial resolution, ii) Soil Moisture and Ocean Salinity Level 3 (SMOS L3; Jacquette *et al*.^130^) at 25 km, iii) European Space Agency Climate Change Initiative (ESA-CCI SM v2.7; Liu *et al*.^131^; Wagner *et al*.^132^) at 25 km, and iv) Global Land Evaporation Amsterdam Model (GLEAM: Martens *et al*.^119^) at 25 km. Recently, the SMAP soil moisture data have been downscaled to a finer spatial resolution of 1 km globally^133,134^ as well as locally^135^. In this study, we focus on the downscaled 1 km SMAP product by Fang *et al*.^133^ while also noting the utility of the other products. Among such limitedly available and disparate observed data are the observations at five locations (Chaiyabhumi, Srisaket, Amnatcharoen, Sakonnakhon, and Bungkan) in Thailand, available at 5-minute intervals from 14th December 2017 to 12th February 2019 and provided by an individual scholar (see Acknowledgment section).

#### Groundwater

Groundwater data in the MRB are collected by respective government agencies in each member country. For example, the National Centre for Water Resources Planning and Investigation (NAWAPI) in Vietnam, the Department of Groundwater Resources (DGR) in Thailand, the Ministry of Water Resources and Meteorology (MOWRAM) in Cambodia, and the Department of Water Resources under the Minister of Natural Resources and Environment (DWR- MONRE) in Laos conduct groundwater monitoring. However, these datasets are generally not available to the public, nor included within the MRC’s database. Some of the datasets (e.g., from NAWAPI) are available for scientific research conducted with an in-country team but are restricted from broader sharing. Further, numerous previous studies have collected groundwater data on an individual basis or obtained from certain partner agencies in the region. Yet, the data have not been shared beyond certain graphics in journal articles. Here, we have digitized all published data, obtainable through our best efforts from published sources^46,54–56,136–156^, and identified various other sources via which groundwater data can be obtained, for example, through formal agreements with respective agencies. Details are provided in Table S1.

### Dam data

Recently, over 100 hydropower dams have been constructed across the MRB, dramatically increasing reservoir storage capacity from ~5 to ~70 km^3^ during 2010–2020^25^. Therefore, dams and their operation have become crucial aspects of hydrologic and ecosystem studies in the MRB, which demand reliable data on the attributes of existing and planned dams as well as on the way reservoirs are operated. Globally, data on large dams are available through the database of the World Register of Dams (WRD), maintained by the International Commission of Large Dams (ICOLD). These data have been synthesized, for example producing theGlobal Reservoir and Dam (GRanD) data^157^ and used in many global studies^112,158–160^. However, these global data include only a few large dams in the MRB, leaving a major information gap regarding the smaller or recently built dams or those that are planned. The GlObal geOreferenced Database of Dams (GOODD; Mulligan *et al*.^161^) includes larger number of dams compared to GRanD and the georeferenced global dams and reservoirs (GeoDAR; Wang *et al*.^162^) and provides richer information on global dams. Yet, the necessary dam attributes (e.g., dam height and reservoir storage capacity) are not comprehensively included in most of these datasets. Recently, Zhang and Gu^163^ developed Global Dam Tracker (GDAT), a comprehensive dam database which includes more than 35,000 global dams with their location, catchment area, and other attributes. The GDAT dataset includes attributes of 466 dams in the MRB. Further, there are notable discrepancies or missing attributes in many of these products (e.g., Shin *et al*.^45^).

In this study, we present the data from the Research Program on Water, Land, and Ecosystems (WLE Mekong; https://wle-mekong.cgiar.org/) as the base product and enhance the database by using information from various other sources. Note that WLE is the primary data source for GDAT for the MRB region. Specifically, building on the efforts of Shin *et al*.^45^, we conducted a thorough inspection of the existing database, made manual corrections using various independent sources (e.g., Google Earth, internet resources on individual dams, published literature and reports), and further verified with credible sources (e.g., Yigzaw *et al*.^164^; Yun *et al*.^165^; Galelli *et al*.^75^; Schmitt *et al*.^166^). We also selected dams that have either or both the dam height and reservoir capacity as these are the two basic attributes for dam impact studies. Finally, we have selected large dams, satisfying on one of the following criteria: (1) dam height ≥ 15 m, (2) storage capacity > 1 million m^3^ (Mm^3^), and (3) installed hydropower capacity > 100 Mega Watts (MW); these large dams are of particular important for basin-wide modeling studies on the impacts of upstream dams on large-scale downstream impacts (e.g., Räsänen *et al*.^167^; Pokhrel *et al*.^9^; Shin *et al*.^45^; Dang *et al*.^47^; Chaudhari and Pokhrel^168^).

### Land use and crop data

Remotely sensed land use and crop data are critical for studies on land use transitions, crop patterns, and hydrologic change. NASA SERVIR Mekong provides land use data for Mekong countries. In particular, the incorporated land use mapping tool within NASA SERVIR Mekong is useful in quantifying the spatial impacts of dams on land use and land cover disturbances^62,169,170^. However, some global products such as Satellite Pour l’Observation de la Terre (SPOT 1–5^171^), Synthetic Aperture Radar (SAR; Balzter *et al*.^172^), Landsat 4–7, Environmental Satellite (ENVISAT) Advanced Synthetic Aperture Radar (ASAR), MODIS, Advanced Very High-Resolution Radiometer (AVHRR), and Sentinel 1–2 satellites are routinely used products for land use change detection in local and global scale studies. Moreover, the European Space Agency- Climate Change Initiative (ESA-CCI) land use data based on the ENVISAT satellite provides continuous annual global data from 1981. Comprehensive information about these land use datasets and satellites is provided in Table 3. Among these land use datasets, we selected ESA-CCI (https://www.esa-landcover-cci.org/) land use data as demonstration in this study owing to its relatively longer temporal coverage and high spatial resolution.

**Table 3:** Details of land use/land cover datasets.

S. No.	Land Use	Satellite	Resolution	Source/ Agency
1	ESRI Land cover	Sentinel- 2	10 m	https://gisgeography.com/living-atlas-of-the-world/
2	Global Land Survey (GLS)	Landsat 7 ETM+	30 m	The University of Maryland with the USGS
3	European Space Agency- Climate Change Initiative (ESA-CCI)	ENVISAT	300 m	http://maps.elie.ucl.ac.be/CCI/viewer/index.php
4	Moderate Resolution Imaging Spectroradiometer (MODIS)	MODIS	500 m	https://modis.gsfc.nasa.gov/data/dataprod/mod12.php; Friedl *et al*.^240^
5	Global Land Cover Characterization (GLCC)	Advanced Very High-Resolution Radiometer	1000 m	Loveland *et al*.^241^
6	GlobeLand30	Landsat	30 m	https://gisgeography.com/usgs-earth-explorer-download-free-landsat-imagery/
7	National Land Cover Database (NLCD)	NOAA- USGS	30 m	https://www.usgs.gov/node/279743
8	SPOT 5	SPOT	20 m	Bartholomé and Belward^171^
9	Synthetic Aperture Radar	SAR	25 m	Balzter *et al*.^172^
10	Copernicus Global Land Service	PROBA- V	100 m	https://land.copernicus.eu/global/lcviewer
11	NASA SERVIR Mekong	Landsat 4–7	30 m	https://landcovermapping.org/en/landcover/

Crop datasets are crucial for accurately modeling hydrological and agricultural processes but datasets on crop types and cropping patterns are not specifically available for the MRB. Thus, as for many other regions, the alternative is to use crop types from Remote sensing. The commonly used Leaf Area Index (LAI) data, an important modelling attribute, in many MRB studies are based on MODIS products (e.g., Son *et al*.^65^; Hu and Mo^173^). Another critical parameter for understanding food security and agricultural productivity is crop yield, which is not available at a basin-wide scale. Therefore, studies in the MRB use global annual data on crop yield such as Food and Agriculture Organization Corporate Statistical Database (FAOSTAT)^76,174,175^. For this study, we obtained country-based annual crop yield data for the period of 1961–2021 for the Lower MRB (LMRB) countries (Cambodia, Laos, Thailand, and Vietnam) from FAOSTAT. The datasets include annual crop yield for crops such as rice, maize, banana, and sugarcane, etc.

Similar to crop yield, crop calendar datasets are available at the global scale. Crop calendar datasets are necessary inputs in hydrological-agricultural modeling, and crucial products for broader agricultural and food security studies. The International Production Assessment Division (IPAD) of the U.S. Department of Agriculture (USDA) Foreign Agricultural Service (FAS) provides global crop calendar data for planting, mid-season, and harvesting periods for grains, oilseeds, and cotton. In addition to IPAD, the Group on Earth Observations Global Agricultural Monitoring (GEOGLAM; Whitcraft *et al*.^176^) has developed crop calendar data using MODIS products for several countries at the national and sub-national scales. Furthermore, Jägermeyr *et al*.^177^ created a gridded dataset of crop calendars for the Global Gridded Crop Model Intercomparison (GGCMI) at a 0.5° spatial resolution. The datasets were generated by combining information from nine observational sources at 0.5° land grid cells for 18 different crops, distinguishing between rainfed and irrigated systems. The dataset includes information on planting day, maturity day, growing season length, primary data source, and the fraction of harvested area. The GGCMI datasets are produced and validated using multiple sources and are gridded products that can be readily used for modeling purposes. We utilized the GGCMI for our study by extracting the MRB region from the global database.

### Irrigated area and Irrigation water use

Irrigation consumes a significant portion of global water withdrawals, accounting for ~70% of total human water use^178^. This is particularly relevant for food security, as a significant portion of global food production (33–40%) is derived from irrigated cropland^179^. Therefore, understanding the spatial distribution of irrigation is essential for managing water resources and ensuring food security; this is crucial in the MRB in light of the growing impacts of climate change and dams on fishery systems (e.g., Sabo *et al*.^77^; Ziv *et al*.^8^; Veldkamp *et al*.^180^) and the potential need for irrigation expansion^76,181^.

However, there are no specific datasets on irrigated areas and irrigation water use for the MRB. As a result, studies on MRB have relied on globally available datasets. The latest version of global maps of irrigated areas provided by Food and Agriculture Organization (FAO) and developed by Siebert *et al*.^182^ are available at 5 arcminutes which has been widely used globally to identify the irrigated area and irrigation water use. Moreover, several studies combined various datasets to generate global maps on irrigated areas (e. g. Zabel *et al*.^183^; Salmon *et al*.^184^; Meier *et al*.^185^). Among different global products, FAO^182^ based irrigated area and irrigation water use data are commonly used in the Mekong. Therefore, we have selected FAO data for this study. However, datasets for Cambodia are missing in global databases. Thus, we acquired the Cambodian census data and subsequently processed these. As a result, the gap in global datasets is filled by processed census data for Cambodia. Though, similar processing can be performed for other countries, however census data is not easily accessible for those countries. Furthermore, ongoing agricultural census surveys in other parts of the basin will be extremely valuable for the research and policy makers.

### Ecological data

#### Nutrients and sediment data

The MRC provides some data on nutrients and sediment, but these datasets are even more sparse than streamflow data and are not freely available. Specifically, the data include Nitrite-Nitrate (NO_3_-N), Total Phosphorous (TP), and Dissolved Oxygen (DO). The MRC Discharge Sediment Monitoring Project (DSMP; Koehnken^186^) that started in 2009 monitors sediment data at certain locations in the downstream regions of the MRB^187^. Sediment concentration estimates are also available from satellite remote sensing, developed by using empirical or physics-based approaches^187,188^. Here, we present and examine the data from the MRC and identify various other data sources.

#### Wetland and inundation data

Accurate wetland datasets are crucial for research on climate change, biodiversity preservation, and the implementation of effective land use policies and wetland conservation strategies. Wetland related studies in the Mekong have primarily used global datasets that are based on satellite observations due to lack of basin wide is-situ data availability. For example, Cho and Qi^70^ used multi-sensor approach to overcome limitations in detecting wetland inundations from 2014 to 2021 in Southeast Asia. Several studies have also identified wetlands in the MRB; however, these are limited to the Mekong Delta^189,190^. On a global scale, Sustainable Wetlands Adaptation and Mitigation Program (SWAMP) wetland maps were produced by Gumbricht *et al*.^191^ which include the wetland categories identified by Ramsar (2013). Furthermore, Tootchi *et al*.^192^ identified global wetlands based on surface water imagery and groundwater constraints. In this study, we provide the comparative evaluation of wetland based on Gumbricht *et al*.^191^ and Tootchi *et al*.^192^ for MRB.

Several studies have used satellite products to generate inundation datasets globally^193–195^. Here, we use the GIEMS-D15 (Global Inundation Extent from Multi-Satellites – Downscaled to 15 arc-seconds; Fluet-Chouinard *et al*.^193^) dataset for inundation maps as the dataset were made available by the authors. Based on topographic indices, the GIEMS-D15 dataset was created by downscaling monthly inundation observations from multiple satellites over a 12-year period from 1993 to 2004^194,195^ to a finer grid resolution of 15 arc-second pixels (~500 m at the equator). However, inundation in the MRB—especially in its downstream regions—is strongly related to precipitation seasonality and flow regulations by dams rather to topography, therefore other methods such as normalized difference vegetation index based flood inundation^196^ than downscaling the data to higher resolution could be more reliable in MRB. Nonetheless, in this study we present the GIEMS-D15 based inundation datasets for the MRB region.

### GHG emission data

Studies on GHG emission in the MRB have focused primarily on emissions from rice cultivation in the Mekong Delta^197,198^. Some studies have investigated alternate farming methods to reduce the GHG emissions in the Delta, but these are rather limited^199–201^. Moreover, a handful of studies have also estimated GHG emissions from hydropower dams in MRB (e.g., Räsänen *et al*.^30^; Shi *et al*.^202^; Wang *et al*.^36^). These studies have produced certain GHG datasets, but a complete timeseries and for the entire MRB is lacking. Therefore, for basin wide studies, global GHG datasets have been used. Global Emissions Database for Global Atmospheric Research (EDGAR; Crippa *et al*.^203^) v4.3.2 is the primary and most reliable source among gridded GHG datasets. The EDGAR dataset compiles anthropogenic emissions data for CO_2_, CH_4,_ and N_2_O based on international statistics and emission factors. Moreover, country specific annual GHG datasets for CO_2_, CH_4_, and N_2_O are also available from Ritchie *et al*.^204^ (OURWORLDINDATA: https://ourworldindata.org/greenhouse-gas-emissions). Here, we employed the EDGAR datasets to infer insights on GHG emissions, which is available at 0.1° (~10 km) spatial resolution and is comprehensive in terms of covering GHG emissions from local and global scales; we consider this dataset as a reliable alternative in the absence of local datasets^205,206^.

### Socio-economic data

Helping advance scientific research and inform science-based management decisions and policymaking for sustainable transboundary basin management requires not only biophysical data (e.g., water, climate, and nutrients), but also socioeconomic data. These data are crucial, for example, to better understand the interactions among climate, water, and societies and ensure food, energy, livelihoods, and water securities under climate change and growing human influence on water systems^1,3,11^. In this study, we synthesize socio-economic data for the four LMRB countries (i.e., Cambodia, Laos, Thailand, and Vietnam), which are obtained from various sources including government websites, the National Institute of Statistics for Cambodia, and the Lao Statistics Bureau for Laos. We further combined these datasets with those available from public repositories such as the OpenDevelopment Mekong (https://opendevelopmentmekong.net/) and the Socioeconomic Data and Applications Center (SEDAC: https://sedac.ciesin.columbia.edu/data/sets/browse). These datasets cover a range of attributes including population demographics, agriculture, gross domestic product, housing, forestry, fishery, road networks, and internal displacement. However, these data are often limited in terms of spatial and temporal coverage, as detailed in Table 4.

**Table 4:** Details of socio-economic datasets synthesized.

S. No.	Countries »	Cambodia	Laos	Thailand	Vietnam	All
1	National sources »	National Institute of Statistic^a^	Lao Statistics Bureau^b^	National Statistics Office (NSO)^c^	General Statistics Office^d^	—
2	Demographics	General Population Census: 1998, 2008, 2019	Lao Population & Housing Census: 2015^e^	See link to NSO	Population: 2011 to 2021	—
		Demographic & Health Survey: 2010, 2021			Population & Housing Census: 2011 to 2021	
		Intercensal Population Survey: 2004, 2013			Health, Culture, Sport, Living standards, Social order, Safety and Environment: 1995 to 2017	
3	Agriculture/Fishing	Census of Agriculture: 2013, 2019	—	See link to NSO	Agriculture, Forestry, Fishery: 2000 to 2021	—
		Intercensal Agriculture Survey: 2019			Rural, Agriculture, and Fishery Census	
4	Environment	—	SDG^f^	See link to NSO	Land & Climate^g^	—
5	Economics	Economic Census: 2011	—	See link to NSO	Employment: 2000 – 2021	—
		Socioeconomic Survey: 2004, 2007, 2009, 2010, 2012, 2013, 2014, 2015, 2016, 2017, 2019				
		Labor Force Survey: 2019				
6	Satellite Imagery Based	—	—	See link to NSO	—	Nighttime Lights^h^
						Global Roads Data^i^
						GRIP Global Roads Data^j^
7	Other	OpenDevelopment Cambodia^k^	OpenDevelopment Laos^l^	OpenDevelopment Thailand^m^	OpenDevelopment Vietnam^n^	SEDAC°
						ASEAN Stats^p^
						APEC Key Economic Indicators^q^
						Global Internal Displacement Database^r^
						SDG Gateway Data^s^

^a^https://www.nis.gov.kh/index.php/km/

^b^https://www.lsb.gov.la/

^c^http://statbbi.nso.go.th/staticreport/page/sector/en/index.aspx

^d^https://www.gso.gov.vn/en/homepage/

^e^https://laosis.lsb.gov.la/tblInfo/TblInfoList.do?rootId=2101000&menuId=2101101&lang=en&keyword=&searchType=undefined

^f^https://www.lsb.gov.la/sdg/en/

^g^https://www.gso.gov.vn/en/administrative-unit-land-and-climate/

^h^https://www.earthdata.nasa.gov/learn/backgrounders/nighttime-lights

^i^https://sedac.ciesin.columbia.edu/data/set/groads-global-roads-open-access-v1

^j^https://www.globio.info/download-grip-dataset

^k^https://opendevelopmentcambodia.net/

^l^https://laos.opendevelopmentmekong.net/

^m^https://thailand.opendevelopmentmekong.net/

^n^https://vietnam.opendevelopmentmekong.net/

°https://sedac.ciesin.columbia.edu/data/sets/browse

^p^https://data.aseanstats.org/

^q^https://statistics.apec.org/index.php/key_indicator/index

^r^https://www.internal-displacement.org/database/displacement-data

^s^https://dataexplorer.unescap.org/.

High resolution gridded population and Gross Domestic Product (GDP) data are key to understanding and better predicting exposure and vulnerability of socioeconomic activities to future climate extremes and developing improved adaptation and mitigation strategies^207^. Gridded population of the World (GPWv4; Doxsey-Whitfield *et al*.^208^) datasets have been extensively used in socioeconomic and environmental studies, such as vulnerability mapping, disaster impacts, and health implications of environmental change^209–211^. However, for our study we used GPWv4 population datasets at 30-arc second (~1 km) spatial resolution and projected population datasets from SEDAC^212^ at 1/8^th^ degree spatial resolution. Furthermore, we utilized gridded GDP data from Kummu *et al*.^24^ at 10-year interval and gridded GDP projections datasets from Wang and Sun^207^ which are consistent with the shared socioeconomic pathways (SSPs). We further provide a comparison between population and GDP projections for all six Mekong countries.

## Data Records

The synthesized datasets are available in the Zenodo repository^213^ (https://zenodo.org/record/7803254). The uploaded datasets are optimized considering user convenience and data size reduction. For example, EM-Earth precipitation and temperature, GLEAM ET, GHG emissions, digitized groundwater, population projections, GDP projections, ground observations of soil moisture, and digitized streamflow datasets are provided in text format. The EM-Earth precipitation and temperature, GLEAM ET, and GHG emissions are gridded datasets with first two rows as locations (longitude and latitude), initial columns as time series (e.g., year, year-month, year-month-day), and rest of the columns as data time series. Moreover, first two columns of populations and GDP projection datasets are gridded locations (longitude and latitude) and the rest of the columns show data for base year or projected years. Digitized groundwater, ground observations of soil moisture, and digitized streamflow data files contain time series in the initial columns and followed by corresponding data in the last column. Data on crop yield, which is country-level annual data, is presented with year in the first column and crop types in the first row; dam attributes (first row) are stored in excel files. GeoTIFF image format is utilized for MODIS ET, irrigated area and irrigation water use, LULC, population, GDP, surface water, and wetland datasets. Soil moisture datasets are stored in MATLAB (.mat) files. Each data folder includes a “Readme” file that provides detailed data description, including the original source, where relevant.

The publicly available datasets such as, satellite precipitation and temperature, ET, surface water, satellite soil moisture, LULC, crop yield, crop calender, wetlands, GHG, and socio-economic datasets are freely available for download from the original sources. Additionally, *in-situ* datasets from the MRC, including precipitation, temperature, wind speed, sunshine hours, specific humidity, streamflow, water level, nutrients, and sediment can be obtained through formal Procedure for Data and Information Exchange and Sharing (PDIES); these data are open to member countries of LMRB and to certain extent the MRC stakeholders^213^.

## Technical validation

### Meteorological data

Among various hydrometeorological datasets identified in methods section, we find the EM-Earth data to be i) relatively inclusive of most climate variables required for analyses and modeling and ii) of reasonable spatial resolution. The dataset also includes multiple ensemble members useful for uncertainty quantification. Thus, we present an analysis of this product, focusing on precipitation and temperature (Fig. 1 and Fig. S1), the two variables of primary interest in many hydrological and ecological studies. Among EM-Earth, APHRODITE, TRMM, IMERGE, Princeton (He *et al*.^100^), and ERA5 precipitation datasets, EM-Earth data show better results when compared against gauge-based data from the MRC at selected locations, except at Kratie (Fig. S1), indicating high accuracy of the ensemble-mean EM-Earth data. Substantial spatial heterogeneity can be seen in precipitation (Fig. 1a) and temperature exhibits a strong north-south gradient (Fig. 1d). In Laos, Vietnam, and the eastern half of Cambodia, annual precipitation is higher compared to other regions in the MRB (Fig. 1a). A higher mean annual temperature in Cambodia, Thailand, and the Mekong Delta was found compared to other parts of the basin (Fig. 1d).

Additionally, we compare the spatial patterns of precipitation and mean temperature for three different datasets: EM-Earth ensemble mean, ERA5, and APHRODITE (Fig. 1a–f), revealing interesting patterns and tendencies. The APHRODITE precipitation was comparatively lower than the other two products in Laos, but the three temperature products display similar spatial patterns across the basin. This suggests that while there may be some differences in the precipitation data, temperature data are more consistent across different sources.

### Hydrological data

#### Streamflow and water level

We evaluate the availability and trends in digitized and MRC-based streamflow and water level at various locations in the MRB. In terms of streamflow and water level data, there are more stations with positive trends than with negative trends (Fig. S2a, b). The alternate positive and negative trends in the streamflow and water level data could be due to seasonal shift in water availability in the streams and different time-period considered to evaluate the trend based on data availability (Fig. S2a, b; Table S2, S3). Moreover, we present the seasonal cycle of streamflow and water level at 8 selected stations across the basin. We find that at all the locations streamflow and water level start increasing from May and peak in August or September (except for Changdu which is peaking in July), following the monsoonal rainfall patterns.

#### ET

**Fig. 2:**
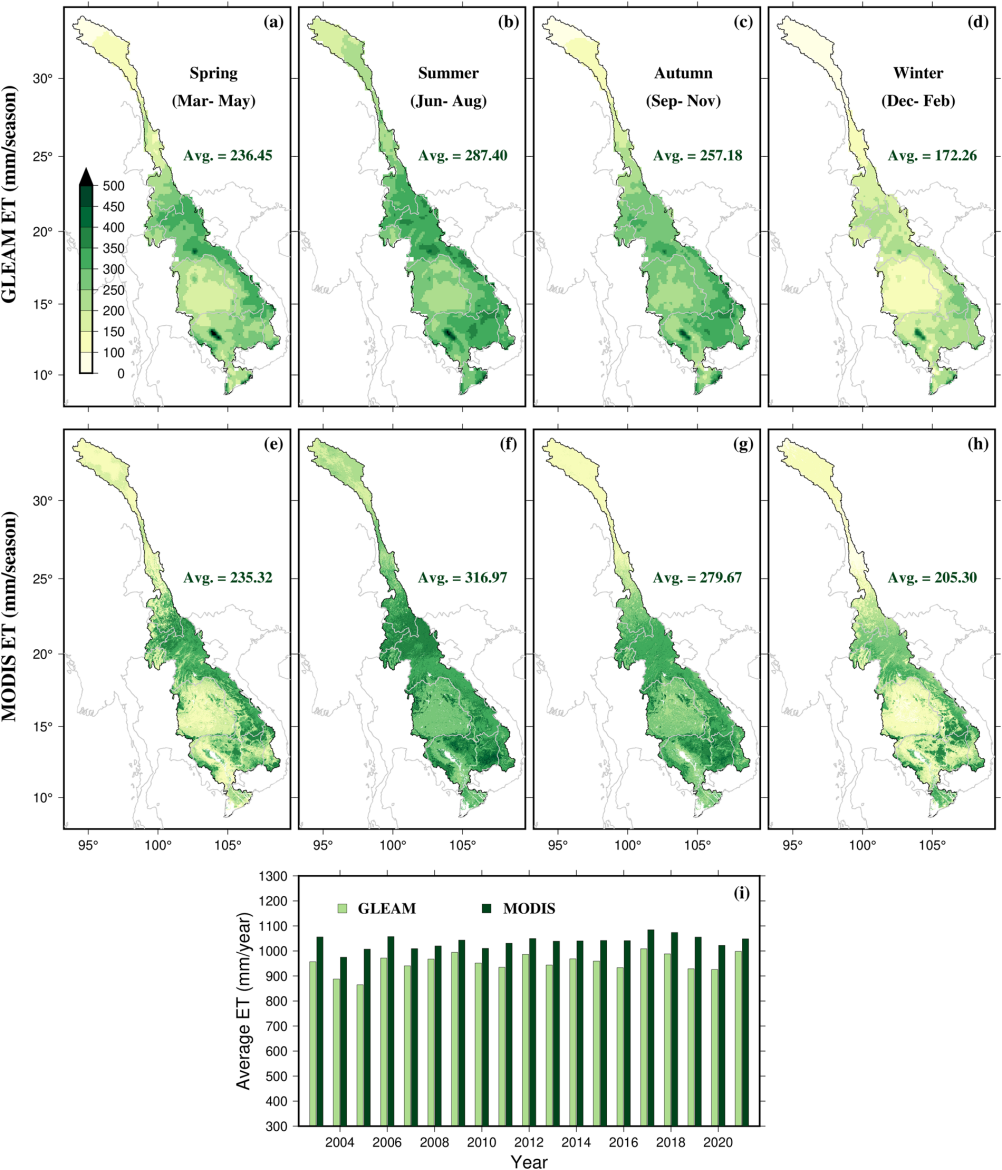
(**a**–**d**) Spatial distribution of seasonal ET from GLEAM data, (**e**–**h**) same as (**a**–**d**) but for MODIS ET, “Ave.” indicates the basin-averaged ET (mm/season), and (**e**) comparison of basin-averaged annual GLEAM and MODIS ET (mm/year).

We compare two ET datasets based on GLEAMv3.6b and MOD16A2GF for four seasons, finding that both datasets show similar spatial pattern across the basin (Fig. 2). Upon analyzing the seasonal pattern in both datasets, it is found that summer season had the highest ET, which is consistent with the seasonal precipitation patterns (Fig. 2a–d). Additionally, we observe that the spatial patterns of ET vary depending on the location within the MRB basin. However, with the exception of the spring season, the seasonal and annual MODIS ET is comparatively higher than GLEAM ET (Fig. 2). Finally, our investigation reveals that the mean annual ET for both datasets for the entire MRB basin exhibit similar increasing trend over time (Fig. 2i). Increased ET in the basin may change the percentage of precipitation that becomes surface water runoff or subsurface recharge which may affect the groundwater levels, groundwater surface water interactions, and soil moisture^214,215^.

#### Surface water

Fig. S3a shows the long-term occurrence of surface water in the MRB based on the data from Ji *et al*.^128^. We highlight two regions of particular interest: one mainly featuring multiple reservoirs, and the other featuring the TSL and Mekong Delta (Fig. S3). For these two regions we compare the surface water based on JRC and Ji *et al*.^128^ (Fig. S3b–e), finding that the data from Ji *et al*.^128^ show lesser extent, and also lower occurrence especially in the Mekong Delta compared to JRC data. Such surface water datasets are crucial for a wide range of studies in the MRB, including for model evaluation and studies on ecological, agricultural, fisheries, and livelihood changes, especially in relation to upstream dam construction. Many recent studies have used these datasets to examine the changing inundation patterns around TSL and Mekong Delta due to climate variability and dam construction^9,29,47^. However, these datasets—mostly satellite based—provide limited information on the changes in surface water such as long-term occurrence or changes in permanent water bodies. Therefore, there are opportunities to develop more accurate and reliable surface water datasets, for example by using information from future satellite missions or improved modeling approaches.

#### Soil moisture

**Fig. 3:**
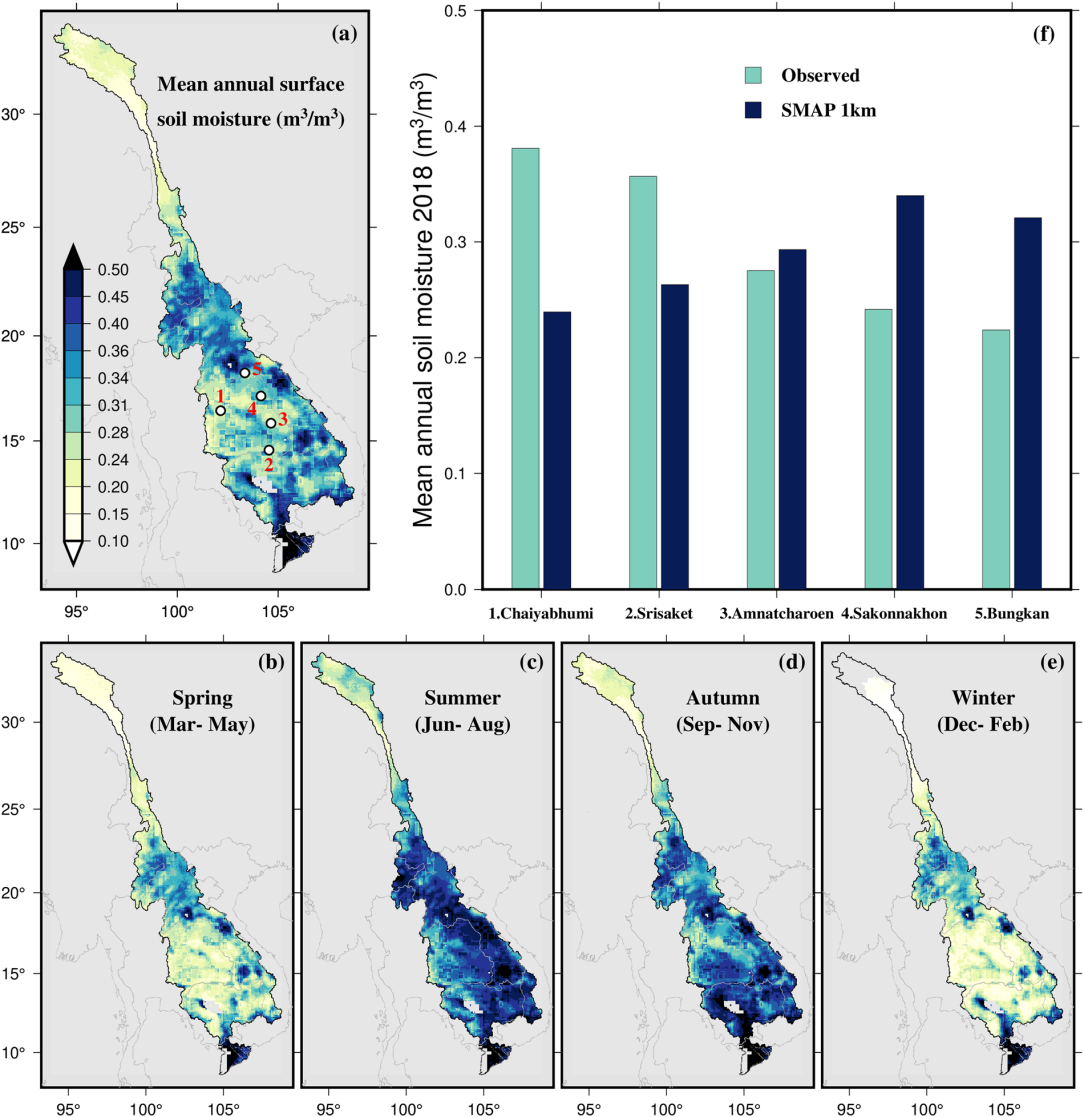
(**a**) Spatial distribution of downscaled SMAP soil moisture data at 1 km spatial resolution for 2016–2021 period. (**b**–**e**) Seasonal variation in the spatial distribution of SMAP data. The grey color indicates ‘no data’. (**f**) Comparison of SMAP data with *in-situ* observation at five locations in Thailand (white circle in panel a).

We chose the soil moisture data from Fang *et al*.^133^ for this study due to its good accuracy and relatively high resolution after reviewing numerous soil moisture products available for the MRB region (discussed in methods section). We analyze the spatial variation of mean annual soil moisture across the MRB and compare downscaled soil moisture data with ground data at 5 locations in Thailand. The mean annual surface (up to 5 cm) soil moisture content in Laos and Vietnam is higher than in other parts of the MRB (Fig. 3a). Mekong Delta in Vietnam and the flood plains in Cambodia show higher soil moisture content. Similarly, the southern parts in the Chinese portion of the MRB, northern Laos, and the subsequent Thailand portions show higher soil moisture. Soil moisture levels are lower in Thailand and some areas of Cambodia that are primarily agricultural (Fig. 3a). A comparison with observed soil moisture at five locations in Thailand suggests that SMAP captures soil moisture content reasonably well (Fig. 3b).

We also analyze the seasonal patterns in the soil moisture data, which reveals that soil moisture is generally higher in the summer and autumn seasons compared to spring and winter (Fig. 3c–f). This pattern is consistent with the typical rainy season in the MRB region, which occurs during the summer and autumn months and results in increased soil moisture levels. We, however, note that this pattern could vary in different regions with different climate patterns that govern seasonal rainfall.

#### Groundwater

**Fig. 4:**
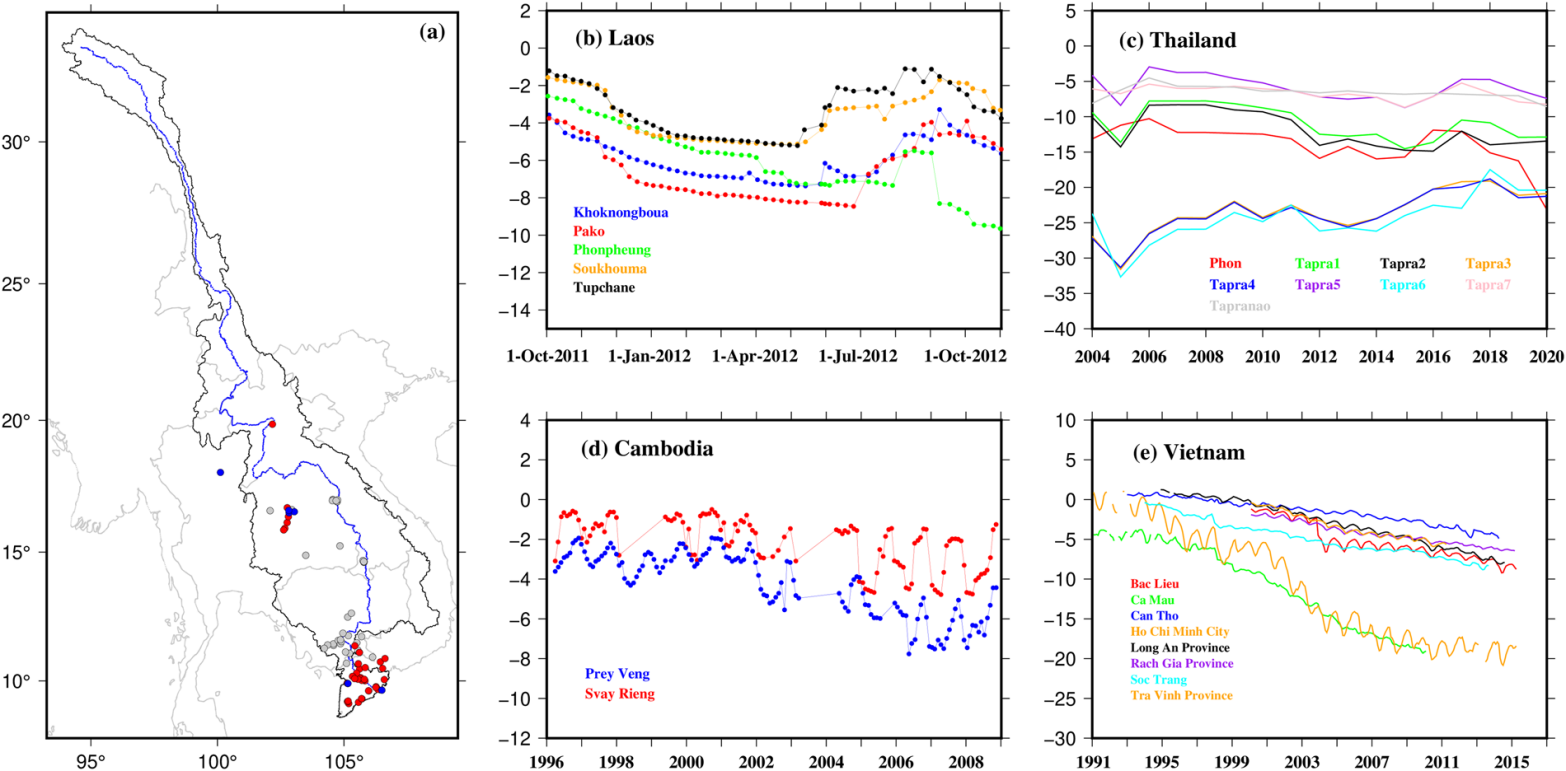
(**a**) Locations of groundwater observation wells at which data are published in the literature. Red and blue colors indicate declining and increasing trends, respectively, in annual groundwater, whereas the grey color indicates either no significant change or groundwater data are available for less than 5 years. We used Mann-Kendall with Sen Slope method to calculate the trend. The time period of available datasets along with trend significance is provided in Table S1, (**b**) daily discrete groundwater depth at 5 locations in Laos from 01-Oct-2011 to 31-Oct-2012, digitized from Vote *et al*.^155^, (**c**) yearly groundwater depth at 7 locations in Thailand from 2004 to 2020, digitized from Muenratch *et al*.^55^, (**d**) daily discrete GW depth from 1996 to 2008 at 2 locations in Cambodia, digitized from Johnston *et al*.^142^, and (**e**) monthly GW depth from 1991–2015 at 7 location in Mekong Delta, Vietnam, digitized from Minderhoud *et al*.^46^.

Groundwater anomalies can be estimated by subtracting the modeled surface water anomalies (e.g., obtained from Global Land Data Assimilation System (GLDAS)) from terrestrial water storage (TWS) anomalies derived from GRACE satellite observations^216,217^. However, the spatial resolution of GRACE data is low, and surface water from GLDAS contains high uncertainty, for example because of missing human interventions. Moreover, observed groundwater datasets for the MRB are not publicly available. Therefore, we present the digitized groundwater data from a series of published literature (Fig. 4). The data consists of temporal measurements of groundwater levels at various locations within the MRB, including daily, monthly, yearly, and seasonal cycles. The highest density of data points was found in the Mekong Delta region, encompassing parts of Vietnam and Cambodia. General examination of the digitized data reveals declining groundwater levels within the MRB, with the most pronounced decreases occurring in the Mekong Delta (Fig. 4). This decreasing trend in groundwater is likely influenced by the high level of groundwater pumping for agricultural purposes in both Vietnam and Cambodia^46,137,146,218^. In addition, the extraction of groundwater for agricultural and domestic use has been linked to subsidence in the Mekong Delta^11,46^. Given these findings, improved management and conservation efforts will be necessary to ensure the sustainable use of groundwater resources in the MRB, particularly in the Mekong Delta region.

### Dam data

**Fig. 5:**
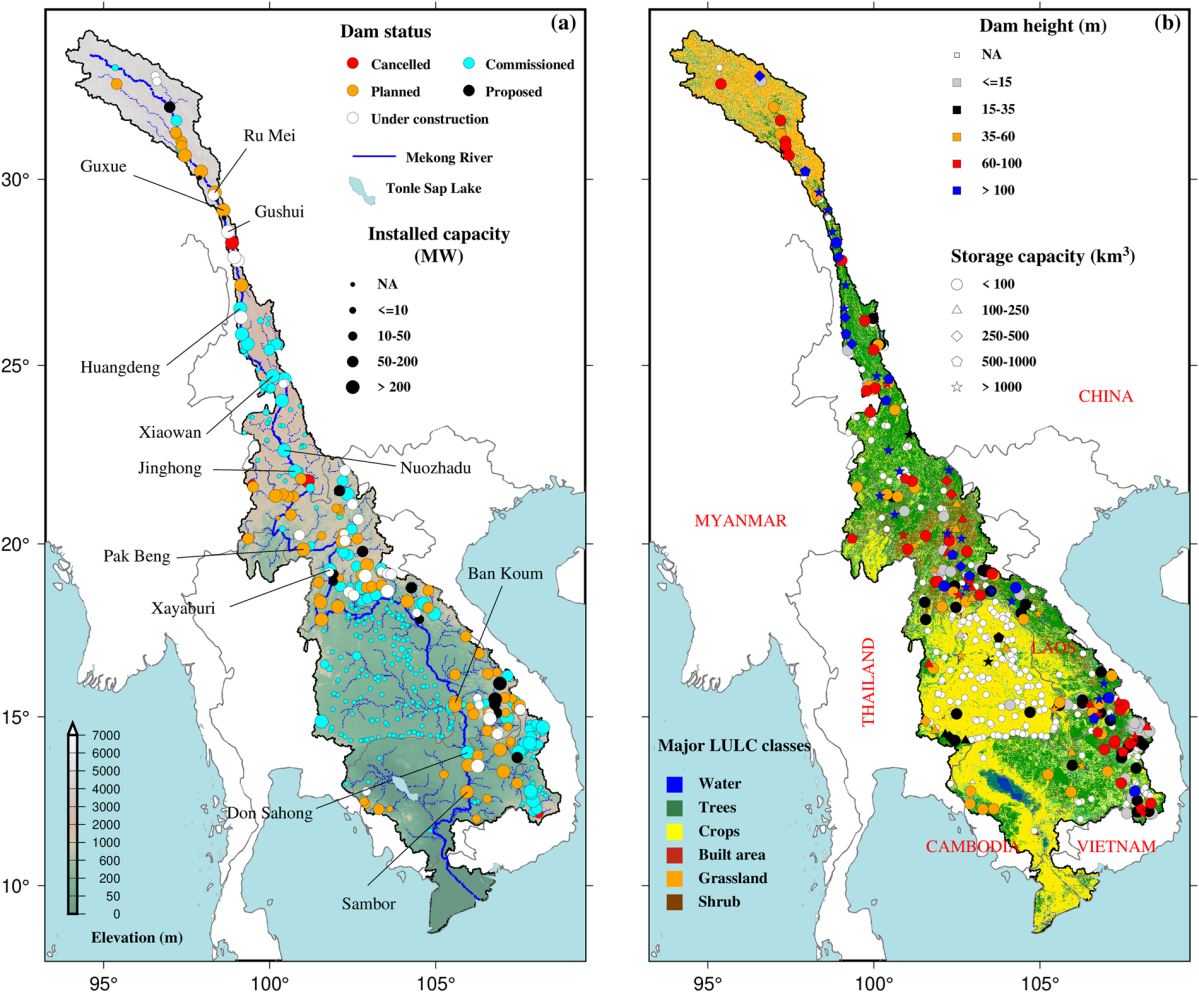
(**a**) Locations of dams in the Mekong River basin. The background image shows elevation (source: Shuttle Radar Topography Mission (SRTM)). Selected large dams—commissioned, under construction, or planned—are labeled. Dam status is color-coded whereas the installed capacity (megawatts) is marked by circle size; NA denotes “data not available”. (**b**) Selected dams, categorized based on dam height and storage capacity; the background image shows land use and land cover (LULC) classes for the year 2020 obtained from ESA-CCI.

Figure 5 depicts the synthesized and corrected dam datasets (see methods section) for the MRB. A more detailed information for the dams selected from the database for hydrological modeling purpose is created, which includes information on dam height, reservoir storage capacity, and reservoir purpose, among others (Table S4). Here we present selected dam attributes such as dam status, installed capacity, dam height, and reservoir storage capacity (Fig. 5). In the past decade, ~100 dams have been constructed in the MRB^25^, with several more currently being planned or under construction, particularly in China, Laos, and Cambodia. The construction of large dams such as Ru Mei, Guxue, Gushui, and Huangdeng in the UMRB (Fig. 5a) has sparked environmental concerns such as the decline in the flood season river flow and annual sediment flux, and water quality deterioration in reservoirs within China^219^. Additionally, the construction of large dams such as Xayaburi, Nuozhadu, and Don Sahong has led to the trapping of sediment flow and disruption of fisheries, raising significant ecological concerns^1,77,220^. Therefore, the dam database is expected to be useful in hydrological, ecological, and socio-economic modelling and consequently future planning and management.

### Land use and crop data

The land use data for the MRB obtained from ESA-CCI were analyzed from 1992 to 2020. Out of eleven land use classes, cropland, tree cover, mosaic tree and shrub, shrubland, and grassland are dominant (Fig. S4). We select two regions in the upper and lower basin for a more in-depth examination (Fig. S4). We found that the LMRB is experiencing a significant increase in crop coverage. In contrast, in the UMRB, there is a substantial increase in tree cover (Fig. S4). In the upper region (region b), there is a slight increase in cropland but a significant increase in tree cover. Tree cover increased primarily from 1996 to 2000, compensating for the loss in shrubland. However, in the lower region (region c), cropland increased substantially with a corresponding decline in tree cover area. Urban areas in region “c” also increased considerably compared to region “b”. Overall, cropland areas in the basin rose steadily until 2012, but started declining since then (Fig. S4c).

To gain further insight into crop dynamics in the LMRB, we analyze data on crop yields for rice, maize, bananas, and sugarcane—the major crops grown in the region—for four LMRB countries (Fig. S5). Our analysis revealed that rice yield has been increasing in all countries, particularly after 1990. Vietnam exhibited the highest rice yield among the four countries. Similarly, maize yield has been increasing across all countries, with Laos exhibiting the highest yield. While banana and sugarcane yields have been decreasing in Cambodia, they have been increasing in the remaining three countries. These data, and the interesting patterns therein, could be useful for studies on water and food security issues in the MRB; however, these datasets are available only at the country level, hence cannot be used for basin-scale analyses or modeling. Nevertheless, the datasets could be used to derive grid-based products through combination with other datasets such as on cropland areas (e.g., Burbano *et al*.^76^).

### Irrigated area and irrigation water use

**Fig. 6:**
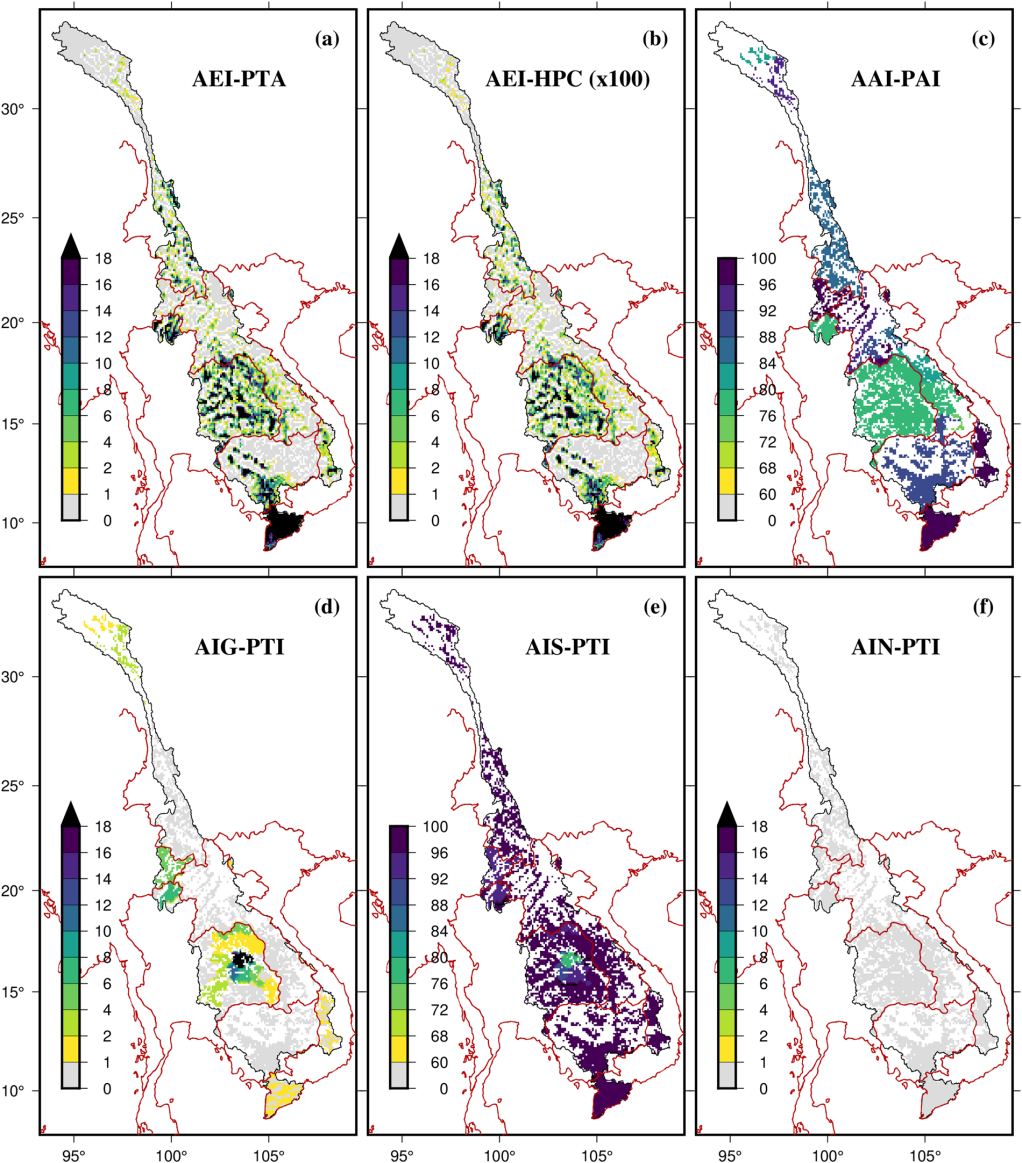
Spatial distribution of (**a**) area equipped for irrigation expressed as a percentage of total area (grid resolution ~ 10 km) (AEI-PTA), (**b**) area equipped for irrigation expressed in hectares per cell (AEI-HPC), (**c**) area actually irrigated expressed as a percentage of area equipped for irrigation (AAI-PAI), (**d**) area irrigated with groundwater expressed as a percentage of total area equipped for irrigation (AIG-PTI), (**e**) area irrigated with surface water expressed as a percentage of total area equipped for irrigation (AIS-PTI), (**f**) area irrigated with water from non-conventional sources expressed as a percentage of total area equipped for irrigation (AIN-PTI).

The areas equipped for irrigation in the MRB mainly ranges from 0 to 20% of the total grid cell area at 0.083333° (~10 km) spatial resolution (Fig. 6a). The Mekong Delta, the flood plains in Cambodia, Thailand, southern part of UMRB, and some portions in Laos are the main areas that are intensively irrigated (Fig. 6b). Results suggest that Vietnam and North Laos portions are more irrigated compared to Cambodia, Thailand, and Southern portion of Laos (Fig. 6c). Except for some portions in Thailand and the Mekong Delta, which are irrigated with groundwater, rest of the basin is irrigated heavily by surface water (Fig. 6d, e). In addition to the conventional sources of irrigation, the use of non-conventional methods for irrigation is extremely limited, as demonstrated in the data presented in Fig. 6f. As the demand for agricultural products from the LMRB is projected to rise by 20–50% in the coming 30 years due to the growing global population^221^, there is a growing risk of food and water insecurity in the basin. To address this issue, it is important to better understand where irrigation is currently happening, what the implications on water and food systems are, and how future irrigation expansion could affect sustainable water use.

**Fig. 7:**
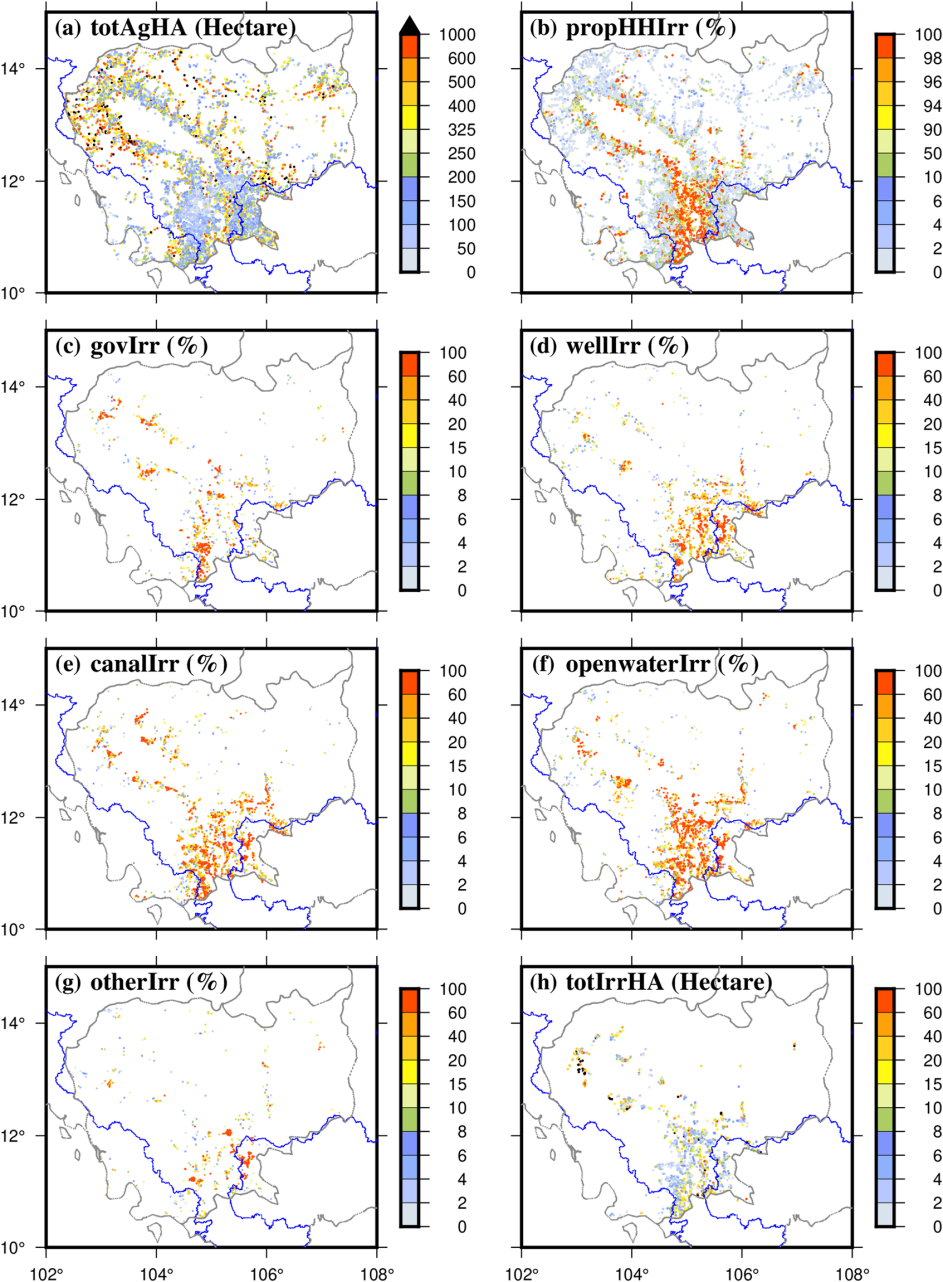
Spatial distribution of irrigated area and irrigation water use datasets collected for Cambodia, (**a**) total hectares in agricultural use (temporary, permanent, meadow/pasture) by village (village size varies) (totAgHA), (**b**) proportion of households by village indicating some use of irrigation (propHHIrr), (**c**) proportion of parcels by village using government owned irrigation (govIrr), (**d**) proportion of parcels by village using well irrigation (wellIrr), (**e**) proportion of parcels by village using canal irrigation (canalIrr), (**f**) proportion of parcels by village using open water irrigation (openwaterIrr), (g) proportion of parcels by village using other types of irrigation (otherIrr), (**h**) total acreage of irrigated land by the village for all type of irrigation (totIrrHA).

To fill irrigation data gaps for Cambodia, we obtained and examined the data from the Cambodian census database, especially focusing on the spatial patterns of irrigation practices (Fig. 7). Results indicate that the highest agricultural land utilization in Cambodia is located in the proximity of the TSL and in flood plain zones (Fig. 7a). Moreover, a higher density of irrigation infrastructure was observed in the flood plain zones when compared to other regions, which was also found by Park *et al*.^146^ (Fig. 7b). Furthermore, the census data also provided insights on the irrigation systems owned and operated by the government. It can be observed that government-owned irrigation systems were less prevalent in comparison to other irrigation practices such as wells, canals, and open water (Fig. 7c–e). Other than well, canal, and open water irrigation, rest of the irrigation sources are insignificant in MRB (Fig. 7g). The total irrigated area data looks incomplete since it is difficult to reconcile responses here with propHHIrr (many NaN values here) (Fig. 7h). Similar census data for irrigation purposes for the other LMRB countries would help in better understanding and modeling the changes in irrigation water use, however these datasets are currently inaccessible.

### Ecological data

#### Nutrients and sediment data

We obtained nutrient datasets (DO, NO_3_-N, and TP) from MRC for our study in MRB as mentioned in methods section. The locations of stations for which data on nutrients (specifically DO, NO_3_-N, and TP) are obtained from the MRC are shown in Fig. S6. We only selected locations for DO, NO_3_-N, and TP where the data was available from 1996–2021. Results indicate that except in Cambodia (near the TSL), DO exhibits a declining trend (Fig. S6a). Further, in the TSL region and Mekong Delta the NO_3_-N concentration has been increasing over time (Fig. S6b). However, TP is increasing across the entire basin except in the Mekong Delta (Fig. S6c). Contrary to the common belief, the construction of multiple dams in the upstream of MRB has increased nutrient concentration downstream^52,222^, especially in Cambodia. Wang *et al*.^223^ also showed an increasing trend in total suspended solids in Cambodia between 2000 to 2018. However, DO and TP show a negative trend in the Mekong Delta. Moreover, nutrients in terms of DO and TP tend to have been discharging to inland water bodies (e.g., lakes) whereas the delivery of these nutrients to the Mekong Delta is declining (Fig. S6).

Similarly, sediment concentration datasets were obtained from the MRC for 18 locations within the MRB (Table S5). However, the datasets are not continuous nor complete for all locations, hence a statistical analysis was not possible. Therefore, we conducted a visual inspection of the data at 10 locations, finding a decline in sediment concentration, particularly at locations within the mainstream Mekong (Figure S7). Only the Mae Suai dam site and Rasi Salai stations, which are not in the mainstream Mekong, show an increase in sediment concentration; the rest of the stations show a decline (Fig. S7). The reduction in sediment load implies downstream impacts including coastal erosion, reduced nutrient supply for aquatic species and agriculture, and land subsidence^22,224^.

#### Wetland and inundation data

**Fig. 8:**
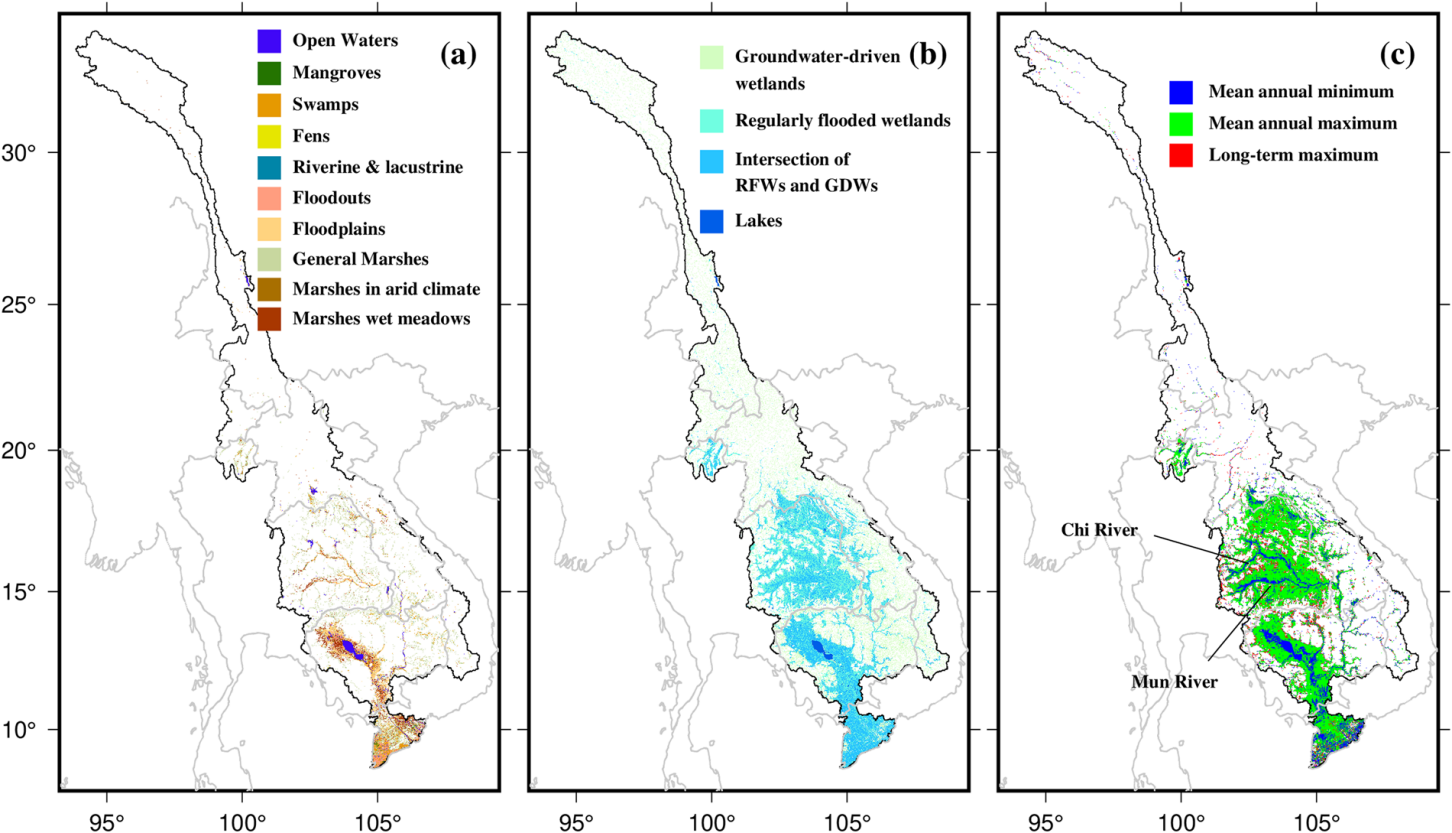
Wetland maps based on (**a**) Gumbricht *et al*.^191^ and (**b**) Tootchi *et al*.^192^; (**c**) GIEMS-D15 map (Fluet-Chouinard *et al*.^193^) inundation extent at 15 arc-second spatial resolution over the basin, with mean annual minimum, mean annual maximum, and long-term maximum inundation area.

We provide a detailed comparison between the two selected wetland products: one based on the maps produced by Gumbricht *et al*.^191^ and the other from Tootchi *et al*.^192^ (Fig. 8a,b). Gumbricht *et al*.^191^ classified the wetlands mainly as open water, mangrove, swamps, fens, riverine and lacustrine, floodplains, and marshes based on geomorphology, moisture condition, and vegetation and soil condition. These wetlands are located mostly in the LMRB with the Mekong Delta housing many swamps, mangroves, and floodouts (Fig. 8a). On the other hand, floodplains of Cambodia and around the lake include open water, marshes, and meadows, etc. (Fig. 8a). However, Tootchi *et al*.^192^ classified the wetlands in two parts which are, i) regularly flooded wetlands (RFW) and ii) groundwater-driven wetlands (GDW). Regularly flooded wetlands were produced by taking the combination of three inundation datasets (ESA-CCI, GIEMS-D15, and JRC surface water). However, groundwater-driver wetlands were derived based on Fan *et al*.^225^ groundwater simulations, considering only pixels with water table depth less than 20 cm. Finally, Tootchi *et al*.^192^ proposed composite wetlands (CW), which are the combinations of RFWs and GDWs (Fig. 8b). However, due to difficulty in downscaling the flooding to higher resolution in MRB, maps based on Tootchi *et al*.^192^ contain higher uncertainty. Therefore, SWAMP data developed by Gumbricht *et al*.^191^ could be considered better product for wetland identification in MRB.

Similarly, the GIEMS-D15 (Fluet-Chouinard *et al*.^193^) dataset were used to identify the annual inundation areas in MRB (Fig. 8c). We find that, the portions of Mekong Delta and TSL basin and the main streamline of Chi and Mun Rivers, tributaries of the Ngun River, were inundated at least every year (Fig. 8c). However, the entire Mekong Delta, a larger portion of the TSL basin, the basins of the Chi and Mun rivers, and areas around Vientiane station were all inundated, considering the annual mean of maximum extent of the flood. Moreover, long-term maximum inundation is almost similar to the mean annual maximum inundation in the MRB. Furthermore, the large uncertainties in the flood inundation data in GIEMS-D15 data propagated from downscaling using DEM data, results in overestimation of the inundation area in MRB (Fig. 8c). Therefore, a basin-wide study at a fine temporal and spatial resolution is essential for the management and conservation of biodiversity and other ecosystem services associated with freshwater.

### GHG emission data

**Fig. 9:**
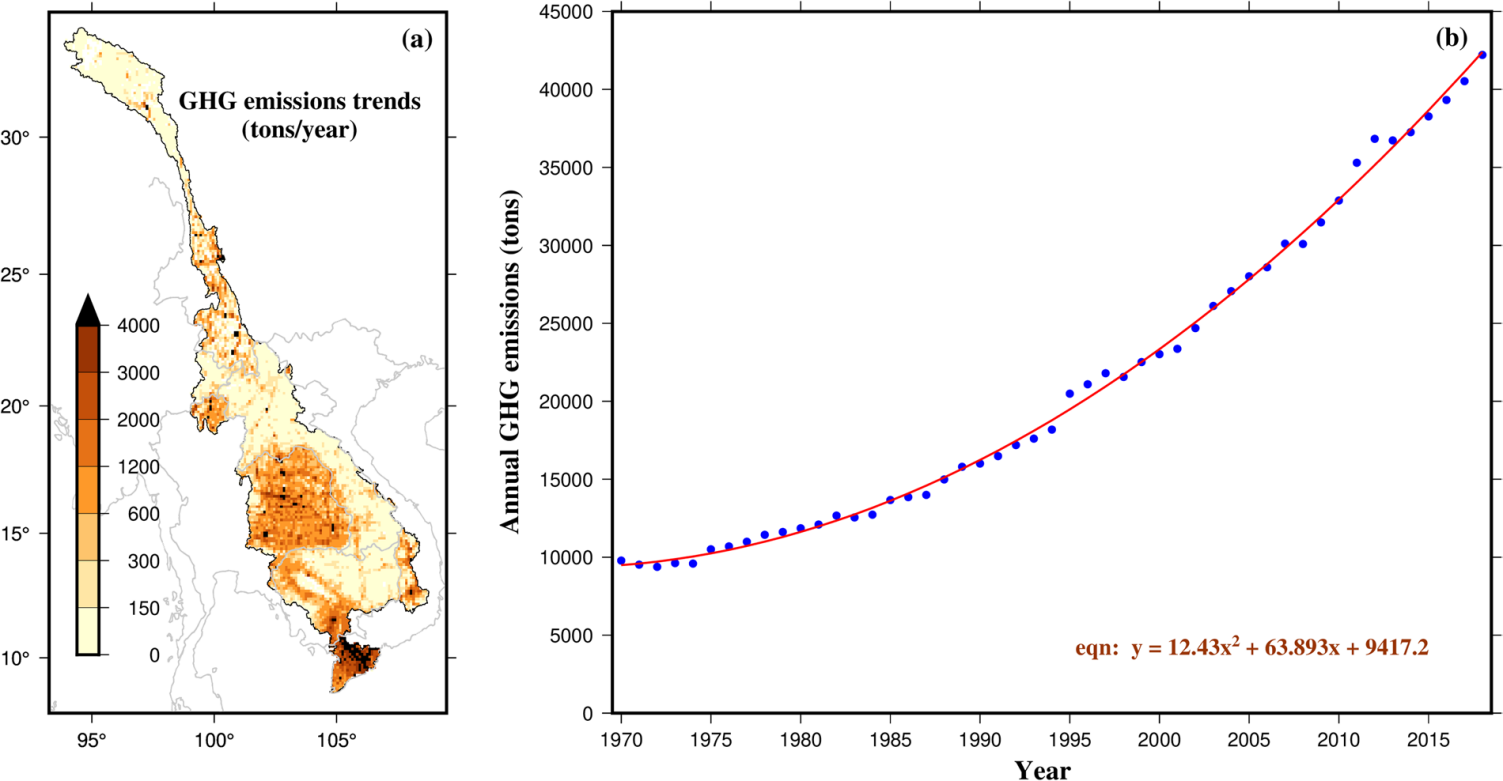
(**a**) Spatial distribution of GHG (CH_4_, CO_2_, and N_2_O) emission rate in tons/year at 0.1° resolution from year 1970–2018, (**b**) timeseries GHG emissions per year from 1970–2018 and trendline fitted with quadratic equation.

The EDGAR based GHG emission datasets cover a substantially long period, enabling a detailed analysis of the spatial patterns of GHG emissions in the MRB. Here, we specifically focus on the trends in GHG emissions over time, with the aim of understanding how these emissions have changed (Fig. 9a). Results indicate a rising trend in GHG emissions throughout the basin. We find a high rate of increase in mean annual GHG emissions in Thailand, the western part of Cambodia (around the TSL), and the Mekong Delta as compared to other regions in the MRB (Fig. 9a). These regions with high GHG emissions having high human population are intensive agricultural regions. Moreover, there is an alarming increase in terms of annual mean GHGs from 1970 to 2018 (Fig. 9b) considering the entire MRB.

### Socio-economic data

**Fig. 10:**
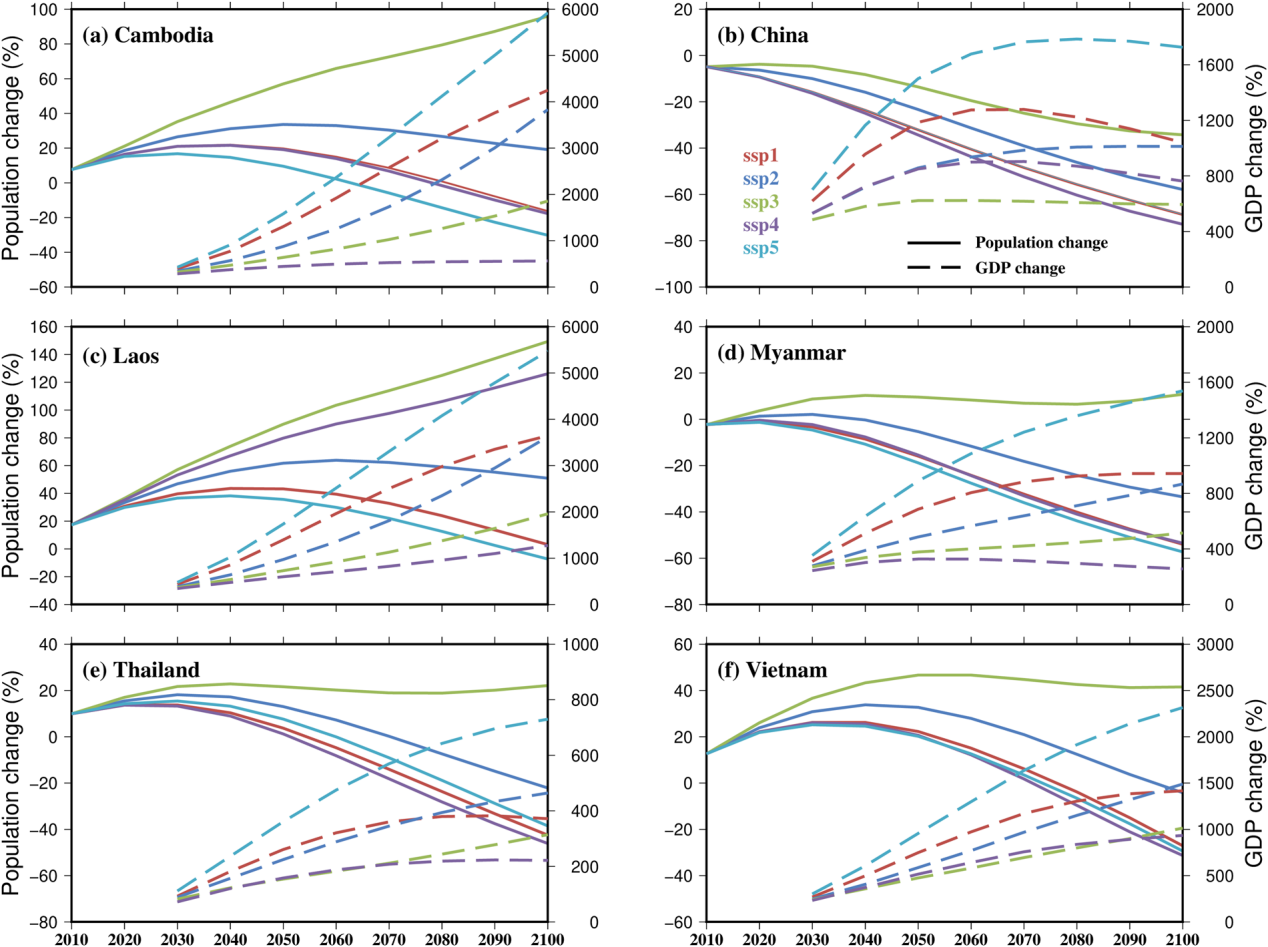
Comparison of projections of population and GDP percentage change for the regions of Cambodia, China, Laos, Myanmar, Thailand, and Vietnam, which come under the MRB from 2010 to 2100. Population change is shown in left side of y-axis and GDP in terms of PPP (USD) change is shown in right side of y-axis with dashed lines.

We compare the projected increase in population and GDP under shared socioeconomic pathways (Fig. 10). Population (2010–2100) and GDP (2030–2100) projections are shown in terms of percentage of the base year as 2000 and 2005, respectively. Projections for the regions of Cambodia, China, Laos, Myanmar, Thailand, and Vietnam, which come within the MRB under shared socioeconomic pathways, were calculated for 5 SSP scenarios which are SSP1 (Sustainability), SSP2 (Middle of the road), SSP3 (Regional rivalry), SSP4 (Inequality), and SSP5 (Fossil-fueled development). In almost all countries, all scenarios show a decrease in the population at the end of the century, except the SSP3 scenario, which shows increasing population in most countries within MRB. Also, the SSP4 and SSP5 scenarios show the most decreasing trend among other. Similarly, there is an increase in the GDP for each country under all five SSP scenarios. Where SSP5 scenario which is projected to have one of the lowest populations shows the highest GDP growth in all countries within MRB. Therefore, population, and GDP are inversely projected in each country of MRB (Fig. 10).

## Usage Notes

The datasets synthesized in this study could form the basis for a range of hydrological, agricultural, ecological, and socioeconomic studies in the MRB. For example, the meteorological datasets from EM-Earth provide a probabilistic approach to meet the diverse requirements of hydrometeorological and ecological applications. The observed climate and streamflow datasets can be used in hydrological models to constrain the streamflow data at sparse locations in the basin. Further, groundwater datasets we digitized from the published literature partly fill the complete vacuum in groundwater data for the MRB. Such data are crucial for groundwater modeling in the MRB, which is indispensable to better understand the rapidly evolving groundwater dynamics across the basin. Indeed, groundwater in the MRB remains relatively poorly studied and needs increased attention. The nutrient datasets at various locations in the MRB could be used to improve the understanding of the changes in water quality as well as to constrain and validate model simulations on riverine nutrient budgets, another research direction that has received very little attention, owing primarily to critical data gaps. Spatial and temporal changes in land use land cover are directly linked to the changes in hydrological, agricultural, and ecological systems across the basin. Thus, the land use data could be of use for a range of hydrological, agricultural, and ecological studies. Moreover, population projections can be used in determining the exposure and vulnerability to future hazards. The synthesized gridded GDP projections will help in identifying the vulnerability, exposure, and resilience of socioeconomic activities under future climate extremes. In summary, the datasets synthesized here are expected to fill the widely acknowledged and long-debated data gap for the MRB, which has hindered socio-hydrological studies—including modeling and analysis—toward improving the understanding of rapidly emerging hydrological, agricultural, and ecological systems within the basin, and providing improved future projections for transboundary water management and sustainability.

## Supplementary information

Supplementary Information

## Data Availability

The codes used for data processing, analysis, and generating results are available at GitHub repository www.github.com/yadupokhrel/Tiwari_Mekong_DataSynthesis_SciData.
