# Supplementary material for: A synthesis of hydroclimatic, ecological, and socioeconomic data for transdisciplinary research in the Mekong
Source: Sci Data. 2023 May 15;10:283. doi: 10.1038/s41597-023-02193-0 (PMC10185688; doi:10.1038/s41597-023-02193-0)
Supplement: Supplementary file 1 — Supplementary Information [file 41597_2023_2193_MOESM1_ESM.docx]

**Supplemental file for**

**A synthesis of hydroclimatic, ecological, and socioeconomic data for transdisciplinary research in the Mekong**

Amar Deep Tiwari^1^, Yadu Pokhrel^1, *^, Daniel Kramer^2^, Tanjila Akhter^1^, Qiuhong Tang^3^, Junguo Liu^4^, Jiaguo Qi^5^, Ho Huu Loc^6^, and Venkataraman Lakshmi^7^

^1^Department of Civil and Environmental Engineering, Michigan State University, East Lansing, Michigan, USA

^2^Department of Fisheries and Wildlife, Michigan State University, East Lansing, Michigan, USA

^3^Key Laboratory of Water Cycle and Related Land Surface Processes, Institute of Geographic Sciences and Natural Resources Research, Chinese Academy of Sciences, Beijing, China

^4^School of Water Conservancy, North China University of Water Resources and Electric Power, Zhengzhou, China

^5^Center for Global Change and Earth Observations, Michigan State University, East Lansing, Michigan, USA

^6^Water Engineering and Management, Asian Institute of Technology, Pathum Thani, Thailand

^7^Engineering Systems and Environment, University of Virginia, Charlottesville, Virginia, USA

*Corresponding Author: Yadu Pokhrel ([ypokhrel@msu.edu](mailto:ypokhrel@msu.edu))

Key Words: *Mekong River basin, data synthesis, climate change, transdisciplinary research, sustainable development*

**Table of contents**

| **Figure/Table** | **Page No.** |
| --- | --- |
| Table S1 | 2-4 |
| Table S2 | 5 |
| Table S3 | 6-7 |
| Table S4 | 7 |
| Table S5 | 10 |
| Fig. S1 | 11 |
| Fig. S2 | 12 |
| Fig. S3 | 10 |
| Fig. S4 | 14 |
| Fig. S5 | 15 |
| Fig. S6 | 16 |
| Fig. S7 | 17 |

Table S1: Locations of boreholes with digitized groundwater dataset details

| **S. No.** | **Data source** | **Station name** | **Longitude** | **Latitude** | **Data availability** |
| --- | --- | --- | --- | --- | --- |
| 1 | Duy et al.^1^ | Cahu Doc (Borehole 14) | 105.1000 | 10.6900 | 1995-2017 |
| 2 | Erban et al.^2^ | P5 | 105.5049 | 11.4948 | Seasonal cycle (Jan-Dec) |
| 3 |  | P9 | 105.6966 | 11.7342 |  |
| 4 |  | P13 | 105.1713 | 11.7706 |  |
| 5 |  | P22 | 105.5525 | 11.1991 |  |
| 6 |  | P29 | 105.2196 | 11.0814 |  |
| 7 |  | S15 | 106.1313 | 10.9346 |  |
| 8 | Fryar et al.^3^ | PN1/11 | 104.7282 | 16.9294 | Daily Jan-2014 to Mar-2015 |
| 9 |  | PN2/16 | 104.7258 | 16.9324 |  |
| 10 | Han et al.^4^ | Well 1-1 | 106.3411 | 9.6719 | qp1 (1991-2011) |
| 11 |  | Well 1-2 | 105.1860 | 9.1130 |  |
| 12 |  | Well 2-3-1 | 105.1271 | 9.9120 | qp2-3 (1991-2016) |
| 13 |  | Well 2-3-2 | 106.4372 | 10.7455 |  |
| 14 |  | Well 2-3-3 | 105.8288 | 10.0064 |  |
| 15 |  | Well 2-3-4 | 105.9789 | 9.6115 |  |
| 16 |  | Well 2-3-5 | 105.5722 | 9.1616 |  |
| 17 | Hoan et al.^5^ | Q401 | 105.1567 | 9.9039 | Monthly timeseries from 2011-2020 for qh, qp3, qp23, qp1, n22 and n21 |
| 18 |  | Q597 | 105.7138 | 9.2974 | Monthly timeseries from 2011-2020 for qp3, qp23, n21 and n13 |
| 19 | IUCN 2011 (ref.^6^) | Q1880 | 105.1395 | 9.1918 | In Ca Mau peninsula based on Phuc 2008 (Thank Nam) |
| 20 |  | Q1770 | 105.143 | 9.1870 |  |
| 21 | Johnston et al.^7^ (IWMI) | Prey Veng | 105.4301 | 11.3778 | 1996-2008 |
| 22 |  | Svay Rieng | 105.6100 | 11.1133 |  |
| 23 | Kabeya et al.^8^ | Tower site | 105.1495 | 12.4910 | 2012-2017 |
| 24 | MRD, JICA^9^  (Kokusai Kogyo Co.) | MEW-01 | NA | NA | 1 year data of the year 1997 |
| 25 |  | MEW-02 | NA | NA |  |
| 26 |  | MEW-03 | NA | NA |  |
| 27 |  | MEW-04 | NA | NA |  |
| 28 |  | MEW-05 | NA | NA |  |
| 29 |  | MEW-06 | NA | NA |  |
| 30 |  | MEW-07 | NA | NA |  |
| 31 |  | MEW-08 | NA | NA |  |
| 32 |  | MEW-09 | 104.5865 | 11.3917 |  |
| 33 |  | MEW-10 | 104.5829 | 11.4486 |  |
| 34 |  | MEW-11 | 104.3542 | 11.4061 |  |
| 35 |  | MEW-12 | 104.2217 | 11.2738 |  |
| 36 |  | MEW-13 | 105.0629 | 11.1320 |  |
| 37 |  | MEW-14 | 104.9606 | 11.8632 |  |
| 38 |  | MEW-15 | 104.7709 | 11.5799 |  |
| 39 |  | MEW-16 | 104.8618 | 11.4566 |  |
| 40 |  | MEW-17 | 104.8960 | 11.5428 |  |
| 41 |  | MEW-19 | 104.8622 | 11.6082 |  |
| 42 |  | MEW-20 | NA | NA |  |
| 43 |  | MEW-21 | NA | NA |  |
| 44 |  | MEW-22 | NA | NA |  |
| 45 |  | MEW-23 | NA | NA |  |
| 46 |  | MEW-24 | NA | NA |  |
| 47 |  | MEW-25 | NA | NA |  |
| 48 |  | MEW-26 | NA | NA |  |
| 49 | Minderhoud et al.^10^ | Rach Gia province | 105.1485 | 9.8988 | 1991-2015 |
| 50 |  | Can Tho | 105.7646 | 10.0351 |  |
| 51 |  | Long An Province | 106.5255 | 10.4882 |  |
| 52 |  | Ho Chi minh City | 106.6223 | 10.8774 |  |
| 53 |  | Bac Lieu | 105.6981 | 9.3082 |  |
| 54 |  | Soc Trang | 105.9714 | 9.6161 |  |
| 55 |  | Tra Vinh province | 106.2677 | 9.7726 |  |
| 56 |  | Ca Mau | 105.1643 | 9.2112 |  |
| 57 | Muenratch et al.^11^ | Tapra 1 | 102.7528 | 16.1380 | Annual mean data from 2004-2020 (Possibility of some uncertainty in locations, however, mean groundwater data of all locations can be considered as mean groundwater level of Khon Kaen district) |
| 58 |  | Tapra 2 | 102.8018 | 16.3619 |  |
| 59 |  | Tapra 3 | 102.8295 | 16.5093 |  |
| 60 |  | Tapra 4 | 103.0400 | 16.5496 |  |
| 61 |  | Tapra 5 | 102.9347 | 16.6278 |  |
| 62 |  | Tapra 6 | 102.8189 | 16.6013 |  |
| 63 |  | Tapra 7 | 102.7571 | 16.7021 |  |
| 64 |  | Tapranao | 102.6636 | 15.9122 |  |
| 65 |  | Phon | 102.6164 | 15.8548 |  |
| 66 | Thu et al.^12^  (Nguyen Thi Thu) | QT1 | 105.6193 | 10.4719 | 2002-2014 |
| 67 |  | QT2 | 105.8371 | 10.5232 |  |
| 68 |  | QT3 | 105.5681 | 10.6727 |  |
| 69 | Nobuhiro et al.^13^ | O Thom | 105.2933 | 12.6433 | 2003-2004 |
| 70 | Park et al.^14^ | P24 | 105.6100 | 11.1133 | 1996-2010 |
| 71 | Petpongpan et al.^15^ | NT 57 | 100.1179 | 18.0416 | 2007-2016 |
| 72 | Le et al.^16^  (Phong VV Le) |  | - | - | Mean GW data of several wells in Mekong delta from 2000-2018 for shallow and deep aquifers |
| 73 | Ribolzi et al.^17^ | Sub-basin outlet | 102.1700 | 19.8558 | 2002-2007 |
| 74 | Seeboonruang et al.^18^ | Well 1 | 104.7453 | 17.0000 | 2010-2012 |
| 75 |  | Well 2 | 104.5605 | 17.0095 |  |
| 76 |  | Well 3 | 104.5588 | 17.0103 |  |
| 77 |  | Well 4 | 104.5613 | 17.0105 |  |
| 78 |  | Well 5 | 104.5393 | 16.9728 |  |
| 79 |  | Well 6 | 104.6888 | 16.9686 |  |
| 80 | Shrestha et al.^19^ | Q219020 | 106.5946 | 10.0531 | 2000-2012 |
| 81 | Tsubo et al.^20^ | Chup Phae District | 102.0800 | 16.5900 | - |
| 82 |  | Surin District | 103.4900 | 14.8800 |  |
| 83 |  | Ubon Ratchathani District | 104.8400 | 15.2400 |  |
| 84 | Van Ty et al.^21^ | BS4 | 105.7210 | 10.0501 | - |
| 85 |  | QT1 | 105.7028 | 10.0241 |  |
| 86 |  | QT8 | 105.6588 | 10.1267 |  |
| 87 |  | QT9 | 105.6153 | 10.1105 |  |
| 88 |  | QT10 | 105.5505 | 10.0688 |  |
| 89 |  | QT11 | 105.5001 | 10.3044 |  |
| 90 |  | QT16 | 105.6450 | 10.1301 |  |
| 91 |  | QT17 | 105.3342 | 10.1774 |  |
| 92 |  | QT18 | 105.4217 | 10.0921 |  |
| 93 | Van et al.^22^ | Q404030-qp3 | 106.2569 | 9.7461 | 2007-2016 |
| 94 |  | Q217030-qp2-3 | 106.4975 | 9.6368 |  |
| 95 | Vote et al.^23^ | Soukhouma | 105.7911 | 14.6481 | 2011-2012 |
| 96 |  | Pako | 105.7491 | 14.6774 |  |
| 97 |  | Khoknongboua | 105.7891 | 14.6110 |  |
| 98 |  | Tupchane | 105.7910 | 14.6282 |  |
| 99 |  | Phonpheung | 105.7836 | 14.6386 |  |
| 100 | Wagner et al.^24^ | Q209 | 105.8033 | 10.0653 | 1993-2011 |

Table S2: Discharge dataset details

| **S. No.** | **Station code** | **Station name** | **longitude** | **latitude** | **Start year** | **End year** |
| --- | --- | --- | --- | --- | --- | --- |
| **1** | KH_014501 | Stung Treng | 105.9502 | 13.5325 | 1910 | 2022 |
| **2** | KH_014901 | Kratie | 106.0176 | 12.4814 | 1924 | 2022 |
| **3** | KH_019801 | Chroy Chang Var | 104.9384 | 11.5874 | 1960 | 2002 |
| **4** | KH_019802 | Kompong Cham | 105.3841 | 11.9110 | 1960 | 2002 |
| **5** | KH_020101 | Phnom Penh Port | 104.9265 | 11.5764 | 1990 | 1990 |
| **6** | LA_011201 | Luang Prabang | 102.1370 | 19.8920 | 1939 | 2018 |
| **7** | LA_011901 | Vientiane | 102.6156 | 17.9310 | 1913 | 2006 |
| **8** | LA_013102 | Thakhek | 104.8067 | 17.3933 | 1924 | 2006 |
| **9** | LA_013401 | Savannakhet | 104.7467 | 16.5617 | 1923 | 2006 |
| **10** | LA_013901 | Pakse | 105.8132 | 15.0998 | 1923 | 2022 |
| **11** | LA_014301 | Ban Chan Noi | 105.8830 | 14.3188 | 1960 | 1964 |
| **12** | LA_230112 | Ban Kok Van | 102.2944 | 19.9542 | 1988 | 2004 |
| **13** | LA_230112 | Nam Ngum Damsite | 102.9200 | 18.8700 | 1966 | 1971 |
| **14** | TH_010501 | Chiang Saen | 100.0885 | 20.2741 | 1960 | 2022 |
| **15** | TH_010601 | Sop Kok | 100.1330 | 20.2420 | 1972 | 1987 |
| **16** | TH_011903 | Chiang Khan | 101.6699 | 17.9003 | 1967 | 2022 |
| **17** | TH_011904 | Pa Mong Dam Site | 102.4264 | 17.9864 | 1968 | 1996 |
| **18** | TH_012001 | Nong Khai | 102.7322 | 17.8814 | 1969 | 2022 |
| **19** | TH_013101 | Nakhon Phanom | 104.7739 | 17.4254 | 1924 | 2022 |
| **20** | TH_013402 | Mukdahan | 104.7332 | 16.5828 | 1923 | 2022 |
| **21** | TH_013801 | Khong Chiam | 105.4935 | 15.3221 | 1966 | 2022 |
| **22** | TH_051001 | Mae Suai Dam Site | 99.5167 | 19.7012 | 1975 | 2001 |
| **23** | TH_370104 | Yasothon | 104.1417 | 15.7817 | 1952 | 2003 |
| **24** | TH_380111 | Pak Mun | 105.4910 | 15.3097 | 1994 | 2000 |
| **25** | TH_380134 | Rasi Salai | 104.1617 | 15.335 | 1979 | 2003 |
| **26** | VN_019804 | My Thuan | 105.9263 | 10.2753 | 2001 | 2007 |
| **27** | VN_039801 | Chau Doc | 105.1335 | 10.7053 | 2001 | 2007 |
| **28** | VN_039803 | Can Tho | 105.7871 | 10.0529 | 2001 | 2007 |
| **29** | VN_980601 | Vam Nao | 105.3634 | 10.5787 | 2001 | 2007 |
| **30** | - | Changdu | 97.1800 | 31.1800 | 1981 | 2000 |
| **31** | - | Gajiu | 100.6000 | 24.4000 | 1961 | 1985 |
| **32** | - | Jiuzhou | 98.9915 | 27.7960 | 1961 | 1985 |
| **33** | - | Yun Jing Hong | 101.3600 | 21.4910 | 1961 | 1985 |

Table S3: Water level dataset details

| **S. No.** | **Station code** | **Station name** | **longitude** | **latitude** | **Start year** | **End year** |
| --- | --- | --- | --- | --- | --- | --- |
| **1** | KH_014501 | Stung Treng | 105.9502 | 13.5325 | 1910 | 2022 |
| **2** | KH_014901 | Kratie | 106.0150 | 12.4767 | 1933 | 2022 |
| **3** | KH_019801 | Chroy Chang Var | 104.9388 | 11.5800 | 1960 | 2012 |
| **4** | KH_019802 | Kompong Cham | 105.3877 | 11.9093 | 1930 | 2022 |
| **5** | KH_020101 | Kg. Thmar (PhnomPenhPort) | 104.9265 | 11.5764 | 1960 | 2022 |
| **6** | KH_020106 | Kompong Luong | 104.2088 | 12.5630 | 1923 | 2022 |
| **7** | KH_550102 | Battambang | 103.2002 | 13.0905 | 1962 | 2012 |
| **8** | KH_580301 | Prey Klong(down) | 103.9136 | 12.1106 | 1994 | 2008 |
| **9** | LA_010802 | Ban Houi Sai | 100.4050 | 20.2820 | 1972 | 1974 |
| **10** | LA_010901 | Pak Beng | 101.1150 | 19.8580 | 1976 | 2022 |
| **11** | LA_011201 | Luang Prabang | 102.1367 | 19.8917 | 1960 | 2022 |
| **12** | LA_011304 | Ban Pakkhone | 101.8550 | 19.4300 | 2001 | 2006 |
| **13** | LA_011401 | Paklay | 101.4130 | 18.2080 | 1960 | 2022 |
| **14** | LA_011901 | Vientiane | 102.6200 | 17.9283 | 1923 | 2022 |
| **15** | LA_012703 | Paksane | 103.6643 | 18.3720 | 1976 | 2022 |
| **16** | LA_013102 | Thakhek | 104.8067 | 17.3933 | 1960 | 2022 |
| **17** | LA_013301 | Keng Kabao | 104.7500 | 16.8130 | 1972 | 1999 |
| **18** | LA_013401 | Savannakhet | 104.7467 | 16.5617 | 1970 | 2022 |
| **19** | LA_013503 | Paktaphane | 105.3500 | 15.9330 | 1995 | 2004 |
| **20** | LA_013901 | Pakse | 105.8132 | 15.0998 | 1960 | 2022 |
| **21** | LA_014101 | Ban Mouang | 105.9120 | 14.9380 | 1974 | 2006 |
| **22** | LA_014303 | Ban Thakho | 105.9866 | 13.9689 | 1995 | 2005 |
| **23** | LA_014304 | Veunkham | 105.9050 | 13.9800 | 1995 | 2006 |
| **24** | LA_120102 | Ban Pak Bak (downstream) | 102.2800 | 19.7430 | 1985 | 2004 |
| **25** | LA_230108 | Veunkham | 102.6170 | 18.1830 | 1991 | 2006 |
| **26** | LA_430107 | Khoueng Sekong | 106.7334 | 15.4334 | 1994 | 2006 |
| **27** | TH_010401 | Sop Ruak | 100.0870 | 20.3480 | 1972 | 2005 |
| **28** | TH_010501 | Chiang Saen | 100.0833 | 20.2733 | 1960 | 2022 |
| **29** | TH_010601 | Sop Kok | 100.1333 | 20.2417 | 1972 | 1993 |
| **30** | TH_010801 | Chiang Khong | 100.3513 | 20.3556 | 1972 | 2022 |
| **31** | TH_011903 | Chiang Khan | 101.6683 | 17.8967 | 1965 | 2022 |
| **32** | TH_011904 | Pa Mong Dam Site | 102.4300 | 17.9850 | 1968 | 2005 |
| **33** | TH_012001 | Nong Khai | 102.7200 | 17.8767 | 1965 | 2022 |
| **34** | TH_012008 | Tha Bo | 102.5920 | 17.8570 | 1980 | 2005 |
| **35** | TH_012301 | Phon Phisai | 103.0780 | 18.0220 | 1972 | 2005 |
| **36** | TH_013101 | Nakhon Phanom | 104.8033 | 17.3983 | 1972 | 2022 |
| **37** | TH_013105 | That Phanom | 104.7330 | 16.9500 | 1972 | 2005 |
| **38** | TH_013402 | Mukdahan | 104.7367 | 16.5400 | 1960 | 2022 |
| **39** | TH_013501 | Khemarat | 105.2000 | 16.0670 | 1965 | 2005 |
| **40** | TH_370104 | Yasothon | 104.1417 | 15.7817 | 1962 | 2003 |
| **41** | TH_380111 | Pak Mun | 105.4950 | 15.3084 | 1981 | 2003 |
| **42** | TH_380134 | Rasi Salai | 104.1617 | 15.3350 | 1979 | 2003 |
| **43** | VN_019803 | Tan Chau | 105.2481 | 10.8006 | 1979 | 2022 |
| **44** | VN_019804 | My Thuan | 105.9264 | 10.2752 | 1960 | 2022 |
| **45** | VN_019805 | My Tho | 106.3570 | 10.3570 | 1985 | 2012 |
| **46** | VN_039803 | Can Tho | 105.7870 | 10.0528 | 1979 | 2022 |
| **47** | VN_980601 | Vam Nao | 105.3632 | 10.5762 | 1985 | 2022 |

Table S4: Attributes of large dams of MRB.

| **S. No.** | **Name** | **Country** | **Commissioned** | **Latitude** | **Longitude** | **Total Storage (MCM)** | **Installed capacity (MW)** | **Dam height (m)** |
| --- | --- | --- | --- | --- | --- | --- | --- | --- |
| 1 | Lower Sesan 2 | CAM | 2018 | 13.551 | 106.264 | 1790 | 480 | 45 |
| 2 | Sambor | CAM | 2020 | 12.787 | 105.939 | 3.794 | 2600 | 56 |
| 3 | Stung Treng | CAM | 2030 | 13.575 | 105.983 | 70 | 980 | 22 |
| 4 | Nam Ngum 1 (Nam Ngum) | LAO | 1971 | 18.531 | 102.55 | 4700 | 148.7 | 75 |
| 5 | Nam Ngum 2 | LAO | 2010/2012 | 18.753 | 102.777 | 3590 | 615 | 181 |
| 6 | Nam Ngum 3 | LAO | 2014/2022 | 19.083 | 102.867 | 979 | 440 | 220 |
| 7 | Nam Ngum 5 | LAO | 2012 | 19.357 | 102.622 | 314 | 120 | 104.5 |
| 8 | Nam Ngieu (Nam Ngiep 3A) | LAO | 2013 | 19.244 | 103.284 | 13.85 | 44 | 31.2 |
| 9 | Nam Ngiep 1 (Important: Re-regulation) | LAO | 2015/2017 | 18.647 | 103.522 | 1200 | 296 | 172 |
| 10 | Nam Ngiep 2 (Nam Sen) | LAO | 2015/2017 | 19.233 | 103.283 | 242.2 | 180 | 70.5 |
| 11 | Nam Ngiep 2A | LAO | 2019 | 19.151 | 106.333 | ROR* | 12.55 | 6 |
| 12 | Nam Ngiep 2B | LAO | 2019 | 19.156 | 103.577 | ROR | 9.44 | 8 |
| 13 | Nam Ngiep 2C | LAO | 2015/2016 | 19.213 | 103.358 | 50 | 45 | 35 |
| 14 | Nam Ou 1 | LAO | 2013/2021 | 20.209 | 102.316 | 10 | 180 | 65 |
| 15 | Nam Ou 2 | LAO | 2015 | 20.387 | 102.451 | 121.7 | 120 | 55 |
| 16 | Nam Ou 3 | LAO | 2013/2021 | 20.721 | 102.673 | 10 | 180 | 72 |
| 17 | Nam Ou 4 | LAO | 2013/2021 | 21.102 | 102.492 | 9.2 | 75 | 47 |
| 18 | Nam Sor | LAO | 2018 | 18.55 | 104.4 | ROR | 7.38 | 0 |
| 19 | Nam Sien Tad Lang | LAO | 2014 | 19.304 | 103.178 | ROR | 5 | 0 |
| 20 | Nam Ou 5 | LAO | 2015 | 21.426 | 102.346 | 335 | 240 | 55 |
| 21 | Nam Ou 6 | LAO | 2015 | 21.779 | 102.195 | 409 | 210 | 108 |
| 22 | Nam Ou 7 | LAO | 2015/2021 | 22.086 | 102.258 | 1134 | 180 | 147 |
| 23 | Nam Leuk (Nam Luek) | LAO | 2000 | 18.434 | 102.949 | 185 | 60 | 46.5 |
| 24 | Nam Lik 1 | LAO | 2014/2016 | 18.619 | 102.387 | 6.8 | 64 | 36.5 |
| 25 | Nam Lik 1n2 (2) | LAO | 2010 | 18.796 | 102.121 | 826 | 100 | 101.4 |
| 26 | Nam Feaung 1 | LAO | 2015 | 18.908 | 101.861 | 30 | 28 | 77 |
| 27 | Nam Feaung 2 | LAO | 2015 | 19.149 | 101.949 | 5 | 25 | 0 |
| 28 | Nam Feaung 3 | LAO | 2015 | 19.257 | 102.094 | 4.8 | 20 | 0 |
| 29 | Nam Sana | LAO | 2014 | 19.205 | 102.311 | ROR | 14 | 7 |
| 30 | Nam Khan 2 | LAO | 2015 | 19.685 | 102.321 | 686.2 | 130 | 155 |
| 31 | Nam Khan 3 | LAO | 2016 | 19.764 | 102.773 | 224 | 60 | 90 |
| 32 | Nam Pha | LAO | 2016/2024 | 20.825 | 100.624 | 2738 | 147.2 | 139.1 |
| 33 | Nam Pha Gnai | LAO | 2020 | 18.989 | 102.827 | ROR | 19.2 | 65 |
| 34 | Nam Long 1 | LAO | 2014 | 20.93 | 100.916 | ROR | 5.6 | 12 |
| 35 | Nam San 3A | LAO | 2015 | 19.143 | 103.67 | ROR | 69 | 28 |
| 36 | Nam San 3B | LAO | 2016 | 19.117 | 103.65 | ROR | 45 | 45 |
| 37 | Nam Mang 1 | LAO | 2015 | 18.534 | 103.196 | 16.52 | 64 | 81.4 |
| 38 | Nam Mang 3 | LAO | 2004/2005 | 18.354 | 102.803 | 49.43 | 40 | 47.9 |
| 39 | Nam Song | LAO | 1996 | 18.798 | 102.427 | ROR | 6 | 21 |
| 40 | Nam Baeng (Nam Beng) | LAO | 2016 | 19.946 | 101.238 | 3611 | 36 | 37.3 |
| 41 | Nam Theun 1 | LAO | 2014/2022 | 18.354 | 104.142 | 2549.2 | 523 | 177 |
| 42 | Nam Nga 1 | LAO | 2017 | 20.281 | 102.207 | 1565.1 | 97.8 | 143 |
| 43 | Nam Nga 2 | LAO | 2017 | 20.4 | 102.017 | ROR | 14.5 | 70.5 |
| 44 | Nam Kong 1 | LAO | 2014/2021 | 14.585 | 106.736 | 505 | 750 | 86.9 |
| 45 | Nam Suang 1 | LAO | 2016/2030 | 20.052 | 102.328 | 87.6 | 40 | 47.3 |
| 46 | Nam Suang 2 | LAO | 2016/2030 | 20.148 | 102.643 | 2014.7 | 134 | 150 |
| 47 | Don Sahong | LAO | 2013/2020 | 13.956 | 105.964 | 115 | 360 | 35.1 |
| 48 | Nam Theun 2 | LAO | 2009/2010 | 17.998 | 104.956 | 3500 | 1075 | 45 |
| 49 | Xe Kaman Xansay | LAO | 2011 | 14.895 | 105.118 | ROR | 32 | 28 |
| 50 | Xe Kaman 1 | LAO | 2011/2016 | 14.963 | 107.152 | 3120 | 322 | 110 |
| 51 | Xe Kaman 3 | LAO | 2009/2015 | 15.436 | 107.337 | 141.5 | 250 | 99 |
| 52 | Xenamnoy 1 | LAO | 2014 | 15.153 | 106.719 | ROR | 14.8 | 7 |
| 53 | Xenamnoy 6 | LAO | 2013 | 15.164 | 106.669 | ROR | 5 | 5 |
| 54 | Xeset 1 | LAO | 1994/1999 | 15.76 | 106.331 | ROR | 45 | 18 |
| 55 | Xeset 3 | LAO | 2016/2020 | 15.345 | 106.308 | 22.89 | 20 | 12 |
| 56 | Xeset 2 | LAO | 2009 | 15.405 | 106.278 | 9.3 | 76 | 26 |
| 57 | XeKatam 1 - XeNamnoy2 | LAO | 2016 | 15.072 | 106.362 | ROR | 20.1 | 0 |
| 58 | Xekong 3A | LAO | 2021 | 15.377 | 106.78 | - | 105 | 41.5 |
| 59 | Xekong 3B | LAO | 2021 | 15.117 | 106.825 | - | 100 | 25.9 |
| 60 | Xekong 4 | LAO | 2014/2020 | 15.513 | 106.788 | 3100 | 300 | 169 |
| 61 | Xekong 5 | LAO | 2020 | 15.974 | 106.931 | 1355.5 | 248 | 213.7 |
| 62 | Salabam (Xelabam) | LAO | 1969/1970 | 15.355 | 105.832 | ROR | 5 | 3.7 |
| 63 | Houay Lamphan | LAO | 2015 | 15.36 | 106.498 | 140 | 88 | 1.038 |
| 64 | Houayho | LAO | 1999 | 14.893 | 106.666 | 3530 | 152.1 | 76.5 |
| 65 | Xepian-Xenamnoy | LAO | 2013/2018 | 15.026 | 106.606 | 885 | 390 | 128 |
| 66 | Theun-Hinboun | LAO | 1998 | 18.261 | 104.562 | 15 | 210 | 27 |
| 67 | Theun-Hinboun Exp | LAO | 2013 | 18.297 | 104.636 | ROR | 220 | 27 |
| 68 | Theun-Hinboun Exp NG8 | LAO | 1970 | 19.821 | 102.104 | 2262 | 60 | 67 |
| 69 | Dak Rung | VN | 2011 | 12.154 | 107.624 | ROR | 8 | 3 |
| 70 | A Luoi (also called A Sap) | VN | 2012 | 16.198 | 107.162 | 60.2 | 170 | 49.5 |
| 71 | Dak Psi 3 | VN | 2010 | 14.798 | 108.007 | ROR | 45 | 0 |
| 72 | Dak Psi 4 | VN | 2010 | 14.768 | 108.003 | ROR | 18 | 0 |
| 73 | Dak Psi 5 | VN | 2012 | 14.661 | 107.937 | 3.53 | 10 | 2 |
| 74 | Dak Ro Sa | VN | 2007 | 14.714 | 107.821 | ROR | 7.5 | 8 |
| 75 | Dak Ne | VN | 2010 | 14.528 | 108.219 | ROR | 8.1 | 0 |
| 76 | Dak Pone | VN | 2009 | 14.576 | 108.305 | ROR | 15.6 | 0 |
| 77 | Dak Po Co | VN | 2015 | 14.655 | 107.808 | ROR | 15 | 0 |
| 78 | Dak Doa | VN | 2010 | 14.185 | 108.107 | 29.13 | 14 | 20 |
| 79 | Dac Me | VN | 2011 | 12.133 | 108.313 | ROR | 5 | 0 |
| 80 | Dak n Teng | VN | 2011 | 12.196 | 107.927 | 25.49 | 13 | 31 |
| 81 | Dak Ru | VN | 2007 | 14.817 | 107.85 | ROR | 7.5 | 6.1 |
| 82 | Dray Hlinh 1 | VN | 1990 | 12.677 | 107.913 | ROR | 12 | 7 |
| 83 | Dray Hlinh 2 | VN | 2007 | 12.677 | 107.913 | ROR | 16 | 7 |
| 84 | Ia Grai 1 | VN | 2012 | 14.022 | 107.65 | ROR | 10.8 | 0 |
| 85 | Ia Grai 2 | VN | 2014 | 13.976 | 107.793 | ROR | 7.5 | 2 |
| 86 | Ia Grai 3 | VN | 2007 | 14.022 | 107.65 | 49.77 | 7.5 | 35.5 |
| 87 | Buon Kuop | VN | 2009 | 12.524 | 107.931 | 73.78 | 280 | 34 |
| 88 | Buon Tua Srah (Buon Tua Sra) | VN | 2009 | 12.285 | 108.048 | 786.9 | 86 | 83 |
| 89 | Plei Krong | VN | 2008 | 14.408 | 107.861 | 1048.7 | 100 | 71 |
| 90 | Upper Kontum (Thuong Kontum) | VN | 2011 | 14.711 | 108.238 | 173.7 | 250 | 73 |
| 91 | Yali | VN | 2001 | 14.223 | 107.793 | 1073 | 720 | 71 |
| 92 | Yan Tann Sien | VN | 2014 | 12.153 | 108.379 | ROR | 19.5 | 0 |
| 93 | Se San 3 | VN | 2006 | 14.217 | 107.7 | 92 | 260 | 96.5 |
| 94 | Se San 3A | VN | 2007 | 14.106 | 107.655 | 80.6 | 96 | 35 |
| 95 | Se San 4 | VN | 2009 | 13.967 | 107.5 | 893 | 360 | 74.1 |
| 96 | Se San 4A | VN | 2008 | 13.933 | 107.466 | 13.13 | 63 | 31.5 |
| 97 | Sre Pok 3 | VN | 2009 | 12.754 | 107.861 | 219 | 220 | 52.5 |
| 98 | Sre Pok 4 | VN | 2009 | 12.873 | 107.781 | 10.1 | 70 | 25 |
| 99 | Sre Pok 4a | VN | 2013 | 12.894 | 198.313 | ROR | 64 | 0 |
| 100 | Krong No 3 | VN | 2016 | 12.212 | 108.285 | 19 | 18 | 28.54 |
| 101 | Krong Kmar | VN | 2008 | 12.452 | 108.337 | ROR | 12 | 88 |
| 102 | Duc Xuyen | VN | CANC | 12.143 | 108.101 | 1749.78 | 58 | 74 |
| 103 | Chulabhorn | THA | 1972 | 16.533 | 101.65 | 165 | 40 | 70 |
| 104 | Huai Kum | THA | 1982 | 16.413 | 101.797 | 22 | 1.2 | 35.6 |
| 105 | Ubol Ratana | THA | 1966 | 16.767 | 102.633 | 2559 | 25.2 | 35.1 |
| 106 | Lam Ta Khong (pumped-storage) | THA | 1974 | 14.865 | 101.56 | 310 | 500 | 40.3 |
| 107 | Nam Pung | THA | 1965 | 16.972 | 103.981 | 165.5 | 6.3 | 41 |
| 108 | Sirindhorn | THA | 1971 | 15.203 | 105.424 | 1967 | 36 | 42 |
| 109 | Pak Mun | THA | 1994 | 15.286 | 105.474 | 225 | 136 | 17 |
| 110 | Guoduo | CHN | 2015 | 31.529 | 97.191 | 82.72 | 160 | 93 |
| 111 | Jinhe | CHN | 2004 | 30.806 | 97.333 | 4.27 | 60 | 34 |
| 112 | Longdi | CHN | 1997 | 26.221 | 99.724 | 13.3 | 10 | 95 |
| 113 | Miaowei | CHN | 2016 | 25.854 | 99.163 | 660 | 1400 | 140 |
| 114 | Gongguoqiao | CHN | 2012 | 25.586 | 99.335 | 316 | 900 | 105 |
| 115 | Xi'er He 1 (downstream Lake Erhai) | CHN | 1979 | 25.579 | 100.202 | ROR | 105 | 30 |
| 116 | Xi'er He 2 | CHN | 1987 | 25.562 | 100.131 | ROR | 50 | 37.25 |
| 117 | Xi'er He 3 | CHN | 1988 | 25.559 | 100.108 | ROR | 50 | 21 |
| 118 | Xi'er He 4 | CHN | 1971 | 25.576 | 100.066 | 14 | 50 | 20 |
| 119 | Lake Erhai | CHN | NA | 25.455 | 100.111 | 2500 | 0 | 11 |
| 120 | XunCun | CHN | 1999 | 25.422 | 99.993 | 73.74 | 78 | 67 |
| 121 | Xiaowan | CHN | 2010 | 24.703 | 100.092 | 14560 | 4200 | 295 |
| 122 | Manwan | CHN | 1992 | 24.625 | 100.446 | 920 | 1670 | 132 |
| 123 | GuaLanZi | CHN | 2016 | 24.492 | 99.814 | 10 | 4.8 | 9.1 |
| 124 | Laoyinyan | CHN | 1997 | 24.469 | 99.818 | 1092 | 16 | 4.2 |
| 125 | ErChahe | CHN | 2008 | 24.437 | 99.859 | 10.92 | 4.8 | 46 |
| 126 | Nan Rong Tian | CHN | 2015 | 24.398 | 99.882 | ROR | 8 | 10.87 |
| 127 | Nanhe 1 | CHN | 2009 | 24.342 | 100.012 | 11.36 | 40 | 56.8 |
| 128 | Nanhe 2 | CHN | 2009 | 24.377 | 100.05 | 12.9 | 25 | 74 |
| 129 | Luozhahe 2 | CHN | 2016 | 24.487 | 100.402 | 3391 | 50 | 71 |
| 130 | Luozhahe 1 | CHN | 2016 | 24.505 | 100.452 | 14.33 | 30 | 59 |
| 131 | Dachaoshan | CHN | 2003 | 24.025 | 100.37 | 890 | 1350 | 111 |
| 132 | Nandeng | CHN | 2010 | 23.704 | 99.889 | 51.49 | 6.4 | 89 |
| 133 | Nuozhadu Dam | CHN | 2014 | 22.656 | 100.418 | 23703 | 5850 | 262 |
| 134 | Jinghong | CHN | 2009 | 22.053 | 100.766 | 1140 | 1750 | 108 |
| 135 | Jinfeng | CHN | 1998 | 21.592 | 101.225 | 19.48 | 16 | 45 |
| 136 | Wapahe Erji | CHN | 2009 | 25.411 | 99.214 | ROR | 5 | 0 |

*ROR= Run-off-the-river

CAM=Cambodia, LAO=Laos, CHN=China, VN=Vietnam, THA=Thailand

Table S5: Sediment concentration details

| **S. No.** | **Station code** | **Station name** | **Longitude** | **Latitude** | **Start year** | **End year** |
| --- | --- | --- | --- | --- | --- | --- |
| 1 | LA_013901 | Pakse | 105.8132 | 15.0998 | 1962 | 2002 |
| 2 | LA_011201 | Luang Prabang | 102.1370 | 19.8920 | 1997 | 1997 |
| 3 | LA_013401 | Savannakhet | 104.7467 | 16.5617 | 2011 | 2019 |
| 4 | LA_014101 | Ban Mouang | 105.9080 | 14.9397 | 1990 | 1990 |
| 5 | LA_120102 | Ban Pak Bak (downstream) | 102.2764 | 19.7442 | 1990 | 2002 |
| 6 | TH_010501 | Chiang Saen | 100.0885 | 20.2741 | 2009 | 2019 |
| 7 | TH_011903 | Chiang Khan | 101.6699 | 17.9003 | 1972 | 1975 |
| 8 | TH_012001 | Nong Khai | 102.7322 | 17.8814 | 1968 | 1971 |
| 9 | TH_013101 | Nakhon Phanom | 104.7739 | 17.4254 | 1967 | 2018 |
| 10 | TH_013402 | Mukdahan | 104.7332 | 16.5828 | 1968 | 1975 |
| 11 | TH_013801 | Khong Chiam | 105.4935 | 15.3221 | 1972 | 2019 |
| 12 | TH_010601 | Sop Kok | 100.1333 | 20.2417 | 1972 | 2015 |
| 13 | TH_010801 | Chiang Khong | 100.4066 | 20.2692 | 1962 | 2019 |
| 14 | TH_011904 | Pa Mong Dam Site | 102.4264 | 17.9864 | 1966 | 2019 |
| 15 | TH_051001 | Mae Suai Dam Site | 99.5167 | 19.7012 | 1975 | 2000 |
| 16 | TH_370104 | Yasothon | 104.1417 | 15.7817 | 1962 | 2003 |
| 17 | TH_380111 | Pak Mun | 105.4910 | 15.3097 | 1972 | 1981 |
| 18 | TH_380134 | Rasi Salai | 104.1617 | 15.3350 | 1979 | 2003 |


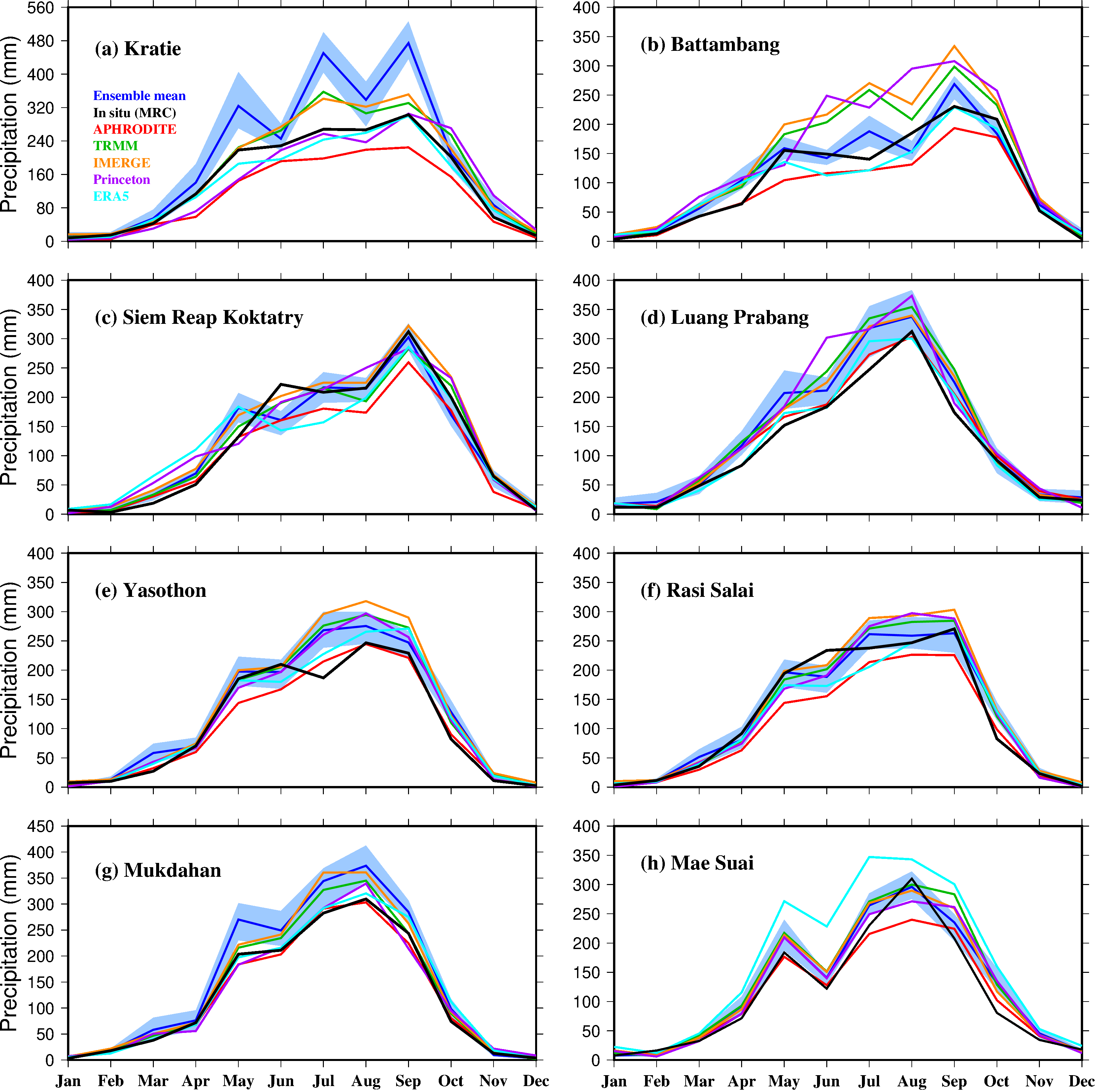


Figure S1: Comparison of ensemble mean (EM-Earth; blue), APHRODITE (red), TRMM (green), IMERGE (orange), Princeton (He et al.,^25^; purple), and ERA5 (cyan) against observed (MRC; black) precipitation dataset at selected locations. Light blue shades show minimum and maximum values of EM-Earth datasets for 25 ensemble members for the period of 2001-2015. The mean precipitation of gridded datasets was calculated by taking the weighted mean of the four nearest grids using the inverse distance method.


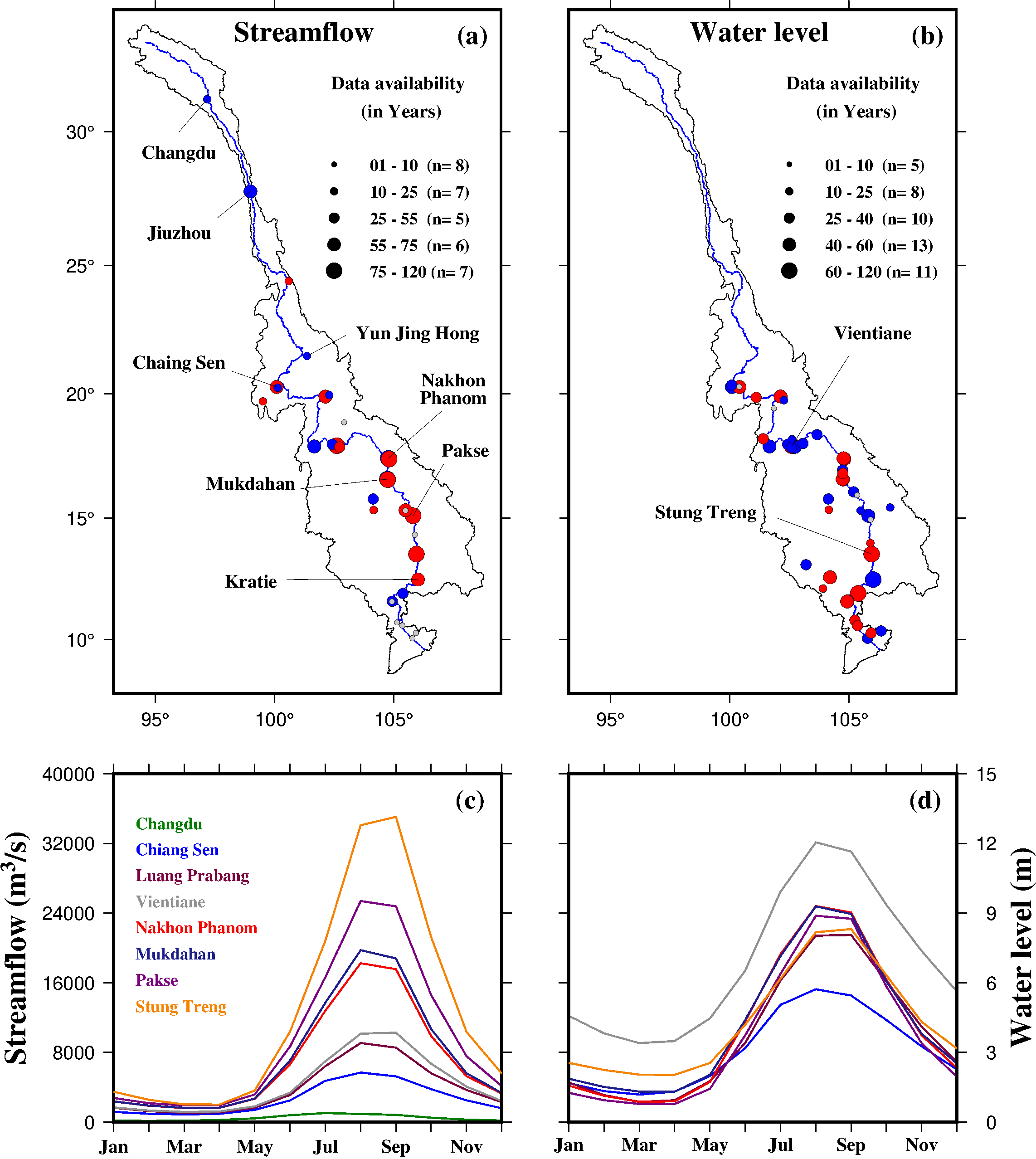


**Figure S2**: Data availability for (a) streamflow and (b) water level shown in the number of years (n). The size of the circle shows the data availability range. Red and blue colors indicate increasing and decreasing trends, respectively, whereas the grey color represents no trend (because of insufficient data to evaluate the trend). The seasonal cycle of streamflow for eight (c) and water level for seven (d) selected stations for the 1981-2000 period. The time period for discharge and water level data availability are presented in Table S2 and Table S3, respectively.


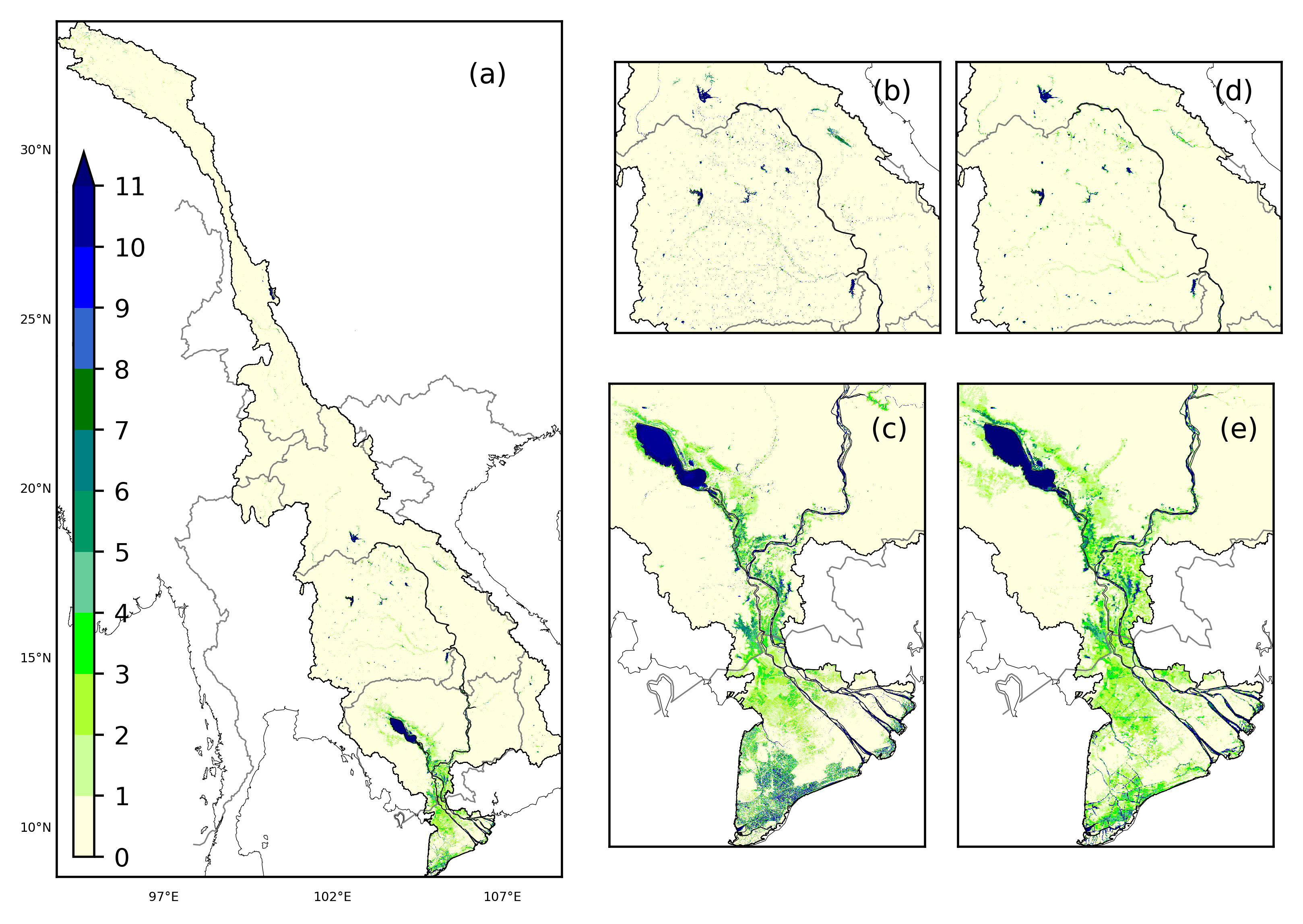


**Figure S3**: (a) Surface water occurrence (2001-2016) in MRB based on Ji et al.^26^ data, (b-c) surface water occurrence magnified at two locations in LMRB based on Pekel et al.^27^ data, (d-e) same as (b-c) but Ji et al.^26^ data.


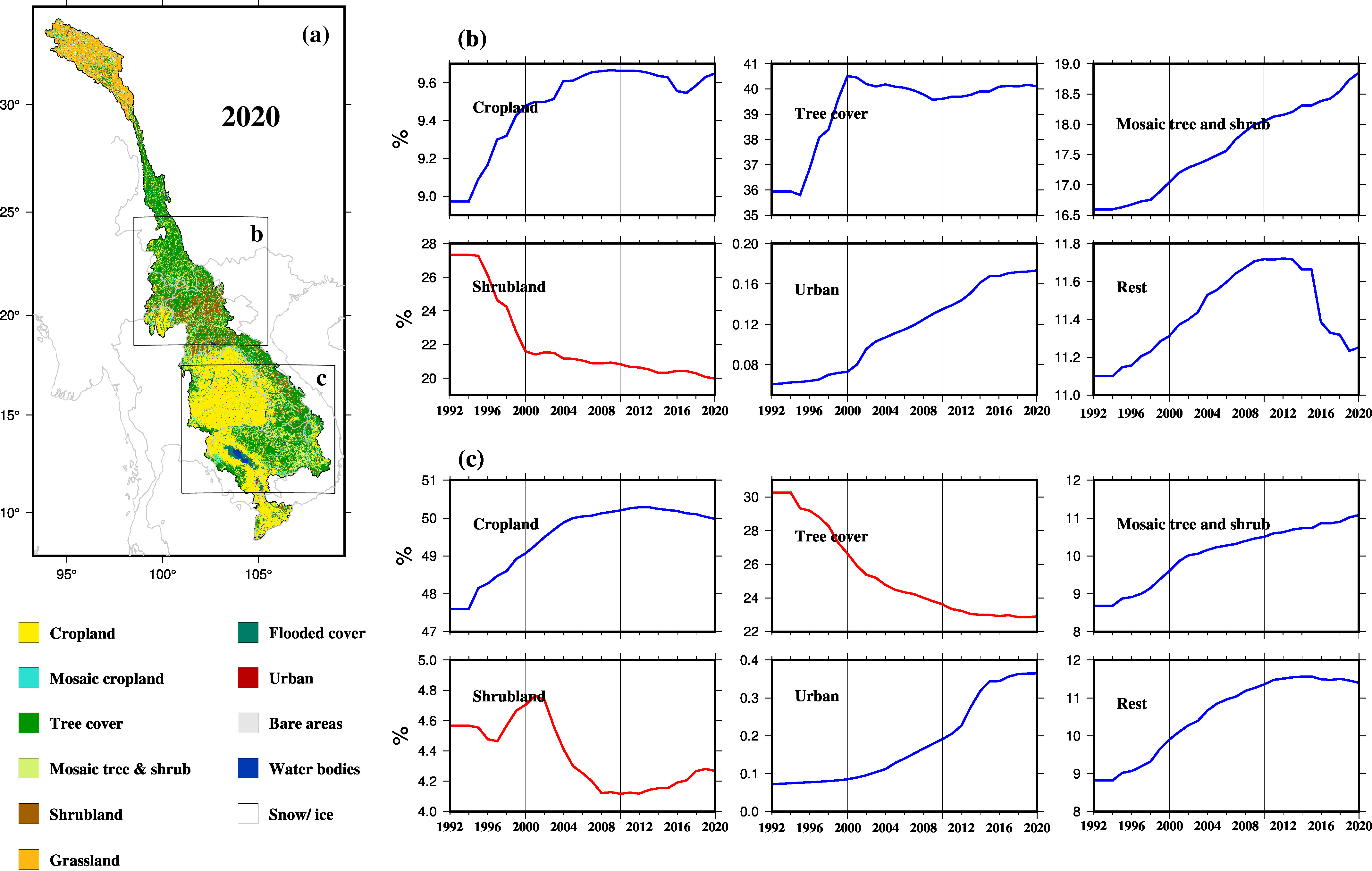


**Figure S4**: Land use and land cover types in the MRB and their historical trends. (a) Land use types for the year 2020. (b-c) Variation in land use for cropland, tree cover, mosaic tree and shrub, shrubland, urban, and rest of the classes from 1992 to 2020 for locations in UMRB and LMRB from 1992 to 2020. Data source: European Space Agency-Climate Impact Initiative (ESA-CCI: <https://www.esa-landcover-cci.org/>; accessed on 10^th^ January 2023). Red color lines show a decreasing trend while blue color lines show an increasing trend.


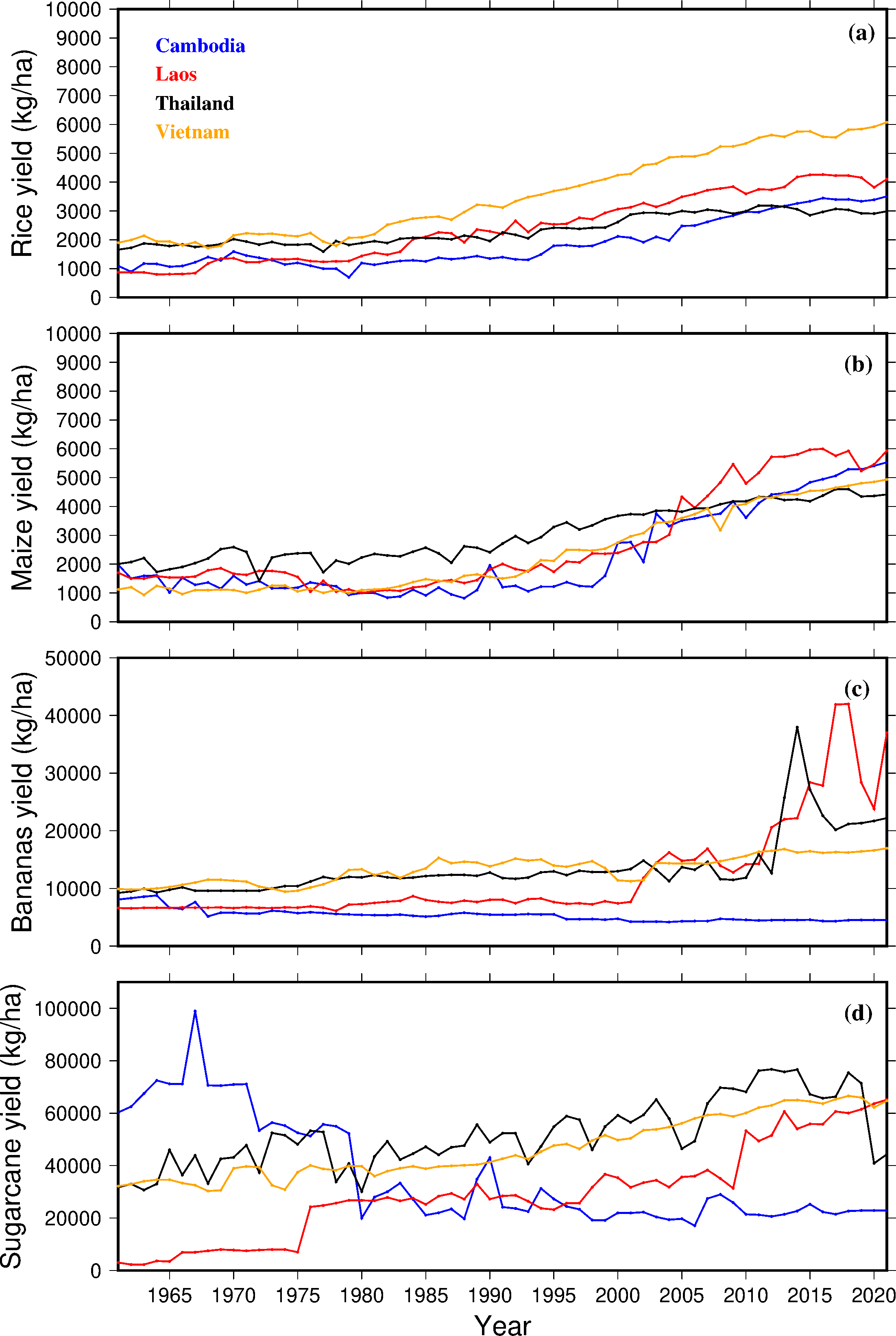


**Figure S5**: Annual time series of the crop (rice, maze, bananas, and sugarcane) yield for four lower MRB countries (Cambodia, Laos, Thailand, and Vietnam) from 1961-2021.


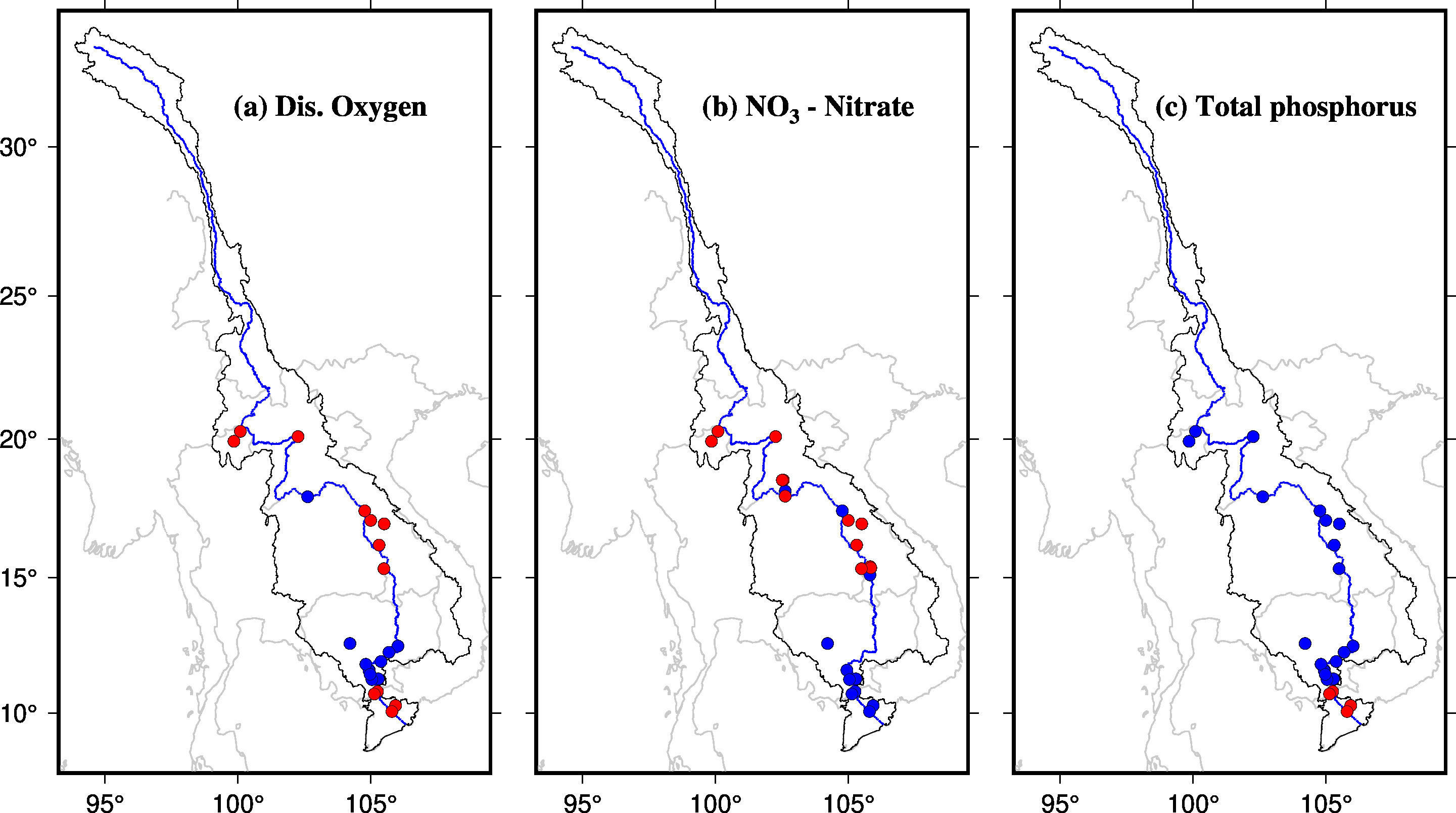


Figure S6: Annual trend in nutrients (a) Dissolved Oxygen (DO), (b) Nitrate (NO_3_), and (c) Total Phosphorous (TP) for the period of 1996-2021. Only those stations were selected where the data was available for the period of 1996-2021. The blue color shows an increasing trend while the red color shows a decreasing trend.


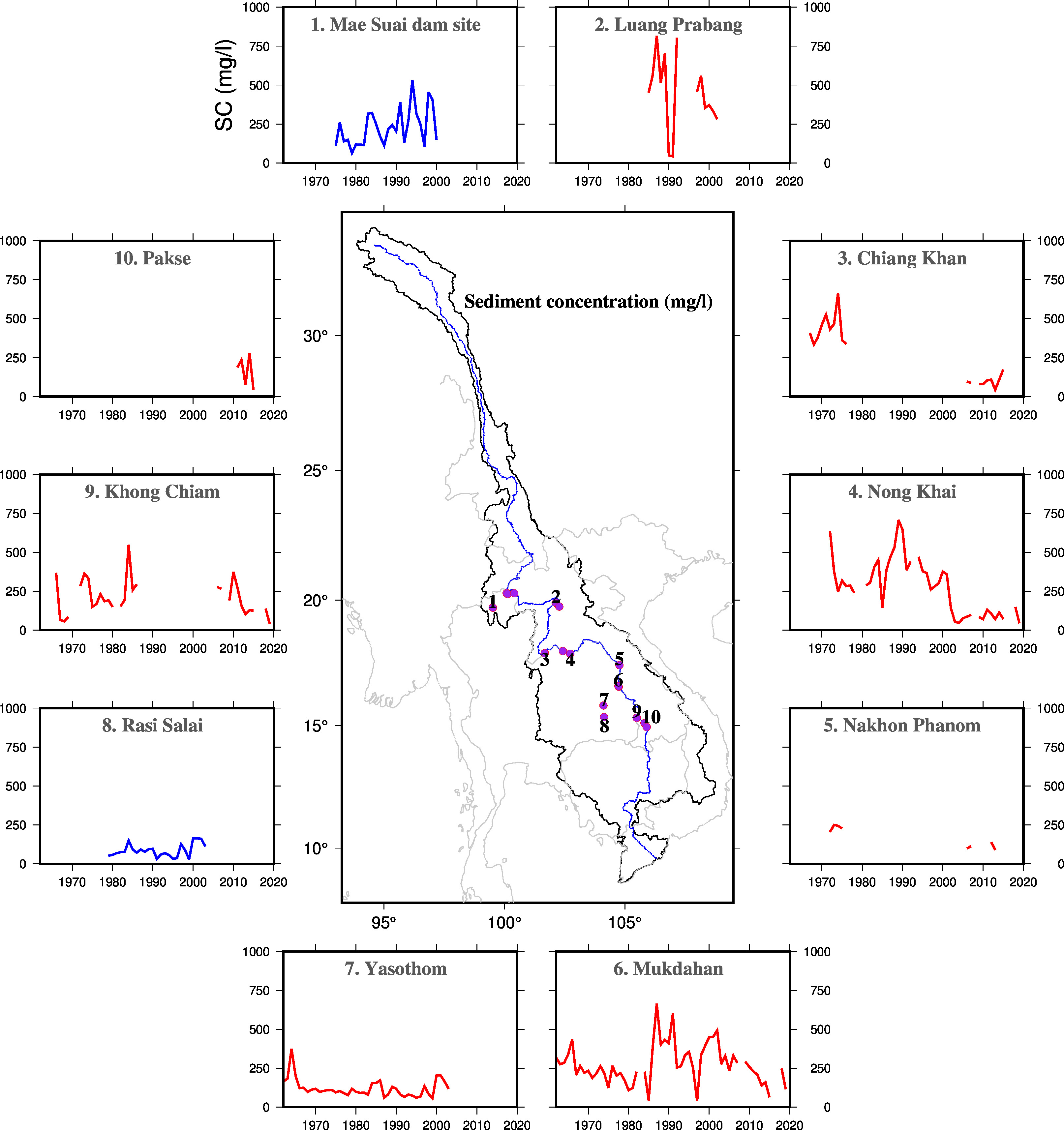


**Figure S7**. Locations of sediment concentration (SC) measurement stations (center map) with annual time-series (sub-plots 1-10) at selected 10 locations marked in the map. Increasing (decreasing) trend is shown with the blue (red) color line.

**References**

1. Duy, N. le *et al.* Groundwater dynamics in the Vietnamese Mekong Delta: Trends, memory effects, and response times. *J Hydrol Reg Stud* **33**, (2021).

2. Erban, L. E. & Gorelick, S. M. Closing the irrigation deficit in Cambodia: Implications for transboundary impacts on groundwater and Mekong River flow. *J Hydrol (Amst)* **535**, 85–92 (2016).

3. Fryar, A. E., Schreiber, M. E., Pholkern, K., Srisuk, K. & Ziegler, B. A. Variability in groundwater flow and chemistry in the Mekong River alluvial aquifer (Thailand): implications for arsenic and manganese occurrence. *Environ Earth Sci* **80**, (2021).

4. Xiao, H. *et al.* Saltwater intrusion into groundwater systems in the Mekong Delta and links to global change. *Advances in Climate Change Research* **12**, 342–352 (2021).

5. Hoan, T. V. *et al.* An Improved Groundwater Model Framework for Aquifer Structures of the Quaternary-Formed Sediment Body in the Southernmost Parts of the Mekong Delta, Vietnam. *Hydrology* **9**, (2022).

6. IUCN. *Mekong Water Dialogues: groundwater in the Mekong Delta*. *International Union for Conservation of Nature* http://scholar.google.com/scholar_lookup?&title=Mekong%20Water%20Dialogues%3A%20groundwater%20in%20the%20Mekong%20Delta&publication_year=2011 (2011).

7. Johnston, R., Roberts, M., Try, T. & de Silva, S. *Groundwater for irrigation in Cambodia*. (2013).

8. Kabeya, N. *et al.* Long-term hydrological observations in a lowland dry evergreen forest catchment area of the lower Mekong River, Cambodia. *Japan Agricultural Research Quarterly: JARQ* **55**, 177–190 (2021).

9. MRD, J. *The study on groundwater development in southern Cambodia: final report*. (2002).

10. Minderhoud, P. S. J. *et al.* Impacts of 25 years of groundwater extraction on subsidence in the Mekong delta, Vietnam. *Environmental Research Letters* **12**, (2017).

11. Muenratch, P., Nguyen, T. P. L., Shrestha, S., Chatterjee, J. S. & Virdis, S. G. P. Governance and policy responses to anthropogenic and climate pressures on groundwater resources in the Greater Mekong Subregion urbanizing cities. *Groundw Sustain Dev* **18**, (2022).

12. Thu, N. T. Groundwater and Surface Water Cycle System in Mekong Delta, Vietnam. *Life and Environmental Sciences* 171 (2017).

13. Nobuhiro, T. *et al.* Evapotranspiration during the late rainy season and middle of the dry season in the watershed of an evergreen forest area, central Cambodia. *Hydrol Process* **22**, 1281–1289 (2008).

14. Park, E. *et al.* Impacts of agricultural expansion on floodplain water and sediment budgets in the Mekong River. *J Hydrol (Amst)* **605**, (2022).

15. Petpongpan, C., Ekkawatpanit, C. & Kositgittiwong, D. Climate change impact on surface water and groundwater recharge in northern Thailand. *Water (Switzerland)* **12**, (2020).

16. Le, P. V. V. *et al.* Responses of groundwater to precipitation variability and ENSO in the Vietnamese Mekong Delta. *Hydrology Research* **52**, 1280–1293 (2021).

17. Ribolzi, O. *et al.* Interacting land use and soil surface dynamics control groundwater outflow in a montane catchment of the lower Mekong basin. *Agric Ecosyst Environ* **268**, 90–102 (2018).

18. Seeboonruang, U. An application of time-lag regression technique for assessment of groundwater fluctuations in a regulated river basin: a case study in Northeastern Thailand. *Environ Earth Sci* **73**, 6511–6523 (2015).

19. Shrestha, S., Bach, T. V. & Pandey, V. P. Climate change impacts on groundwater resources in Mekong Delta under representative concentration pathways (RCPs) scenarios. *Environ Sci Policy* **61**, 1–13 (2016).

20. Tsubo, M. *et al.* Effects of soil clay content on water balance and productivity in rainfed lowland rice ecosystem in Northeast Thailand. *Plant Prod Sci* **10**, 232–241 (2007).

21. van Ty, T. *et al.* Spatiotemporal variations in groundwater levels and the impact on land subsidence in CanTho, Vietnam. *Groundw Sustain Dev* **15**, (2021).

22. Van, T. P. & Koontanakulvong, S. Estimation of groundwater use pattern and distribution in the coastal mekong delta, Vietnam via socio-economical survey and groundwater modelling. *Engineering Journal* **23**, 487–499 (2019).

23. Vote, C. *et al.* The use of groundwater as an alternative water source for agricultural production in southern Lao PDR and the implications for policymakers. in *A policy dialogue on rice futures: rice-based farming systems research in the Mekong region* 103–115 (2014).

24. Wagner, F., Tran, V. B. & Renaud, F. G. Groundwater Resources in the Mekong Delta: Availability, Utilization and Risks. in *The Mekong Delta System* 201–220 (Springer, 2012).

25. He, X., Pan, M., Wei, Z., Wood, E. F. & Sheffield, J. A global drought and flood catalogue from 1950 to 2016. *Bull Am Meteorol Soc* **101**, E508–E535 (2020).

26. Ji, L., Gong, P., Wang, J., Shi, J. & Zhu, Z. Construction of the 500-m Resolution Daily Global Surface Water Change Database (2001–2016). *Water Resour Res* **54**, 10,270-10,292 (2018).

27. Pekel, J. F., Cottam, A., Gorelick, N. & Belward, A. S. High-resolution mapping of global surface water and its long-term changes. *Nature* **540**, 418–422 (2016).
